# 17q21 asthma-risk variants switch CTCF binding and regulate IL-2 production by T cells

**DOI:** 10.1038/ncomms13426

**Published:** 2016-11-16

**Authors:** Benjamin Joachim Schmiedel, Grégory Seumois, Daniela Samaniego-Castruita, Justin Cayford, Veronique Schulten, Lukas Chavez, Ferhat Ay, Alessandro Sette, Bjoern Peters, Pandurangan Vijayanand

**Affiliations:** 1 La Jolla Institute for Allergy and Immunology, La Jolla, California 92037, USA; 2 German Cancer Research Center (DKFZ), Division of Pediatric Neurooncology, 69120 Heidelberg, Germany; 3 Clinical and Experimental Sciences, National Institute for Health Research Southampton Respiratory Biomedical Research Unit, University of Southampton, Faculty of Medicine, Southampton SO16 6YD, UK

## Abstract

Asthma and autoimmune disease susceptibility has been strongly linked to genetic variants in the 17q21 haploblock that alter the expression of *ORMDL3*; however, the molecular mechanisms by which these variants perturb gene expression and the cell types in which this effect is most prominent are unclear. We found several 17q21 variants overlapped enhancers present mainly in primary immune cell types. CD4^+^ T cells showed the greatest increase (threefold) in *ORMDL3* expression in individuals carrying the asthma-risk alleles, where ORMDL3 negatively regulated interleukin-2 production. The asthma-risk variants rs4065275 and rs12936231 switched CTCF-binding sites in the 17q21 locus, and 4C-Seq assays showed that several distal *cis*-regulatory elements upstream of the disrupted *ZPBP2* CTCF-binding site interacted with the *ORMDL3* promoter region in CD4^+^ T cells exclusively from subjects carrying asthma-risk alleles. Overall, our results suggested that T cells are one of the most prominent cell types affected by 17q21 variants.

## Abstract (web summary)

Variations in the 17q21 locus are linked to asthma susceptibility and other autoimmune diseases. Here, the authors perform cell type-specific functional genomic analyses of asthma-risk SNPs, and show a genotype specific mechanism of differential gene regulation relevant to immune function.

## Graphical abstract

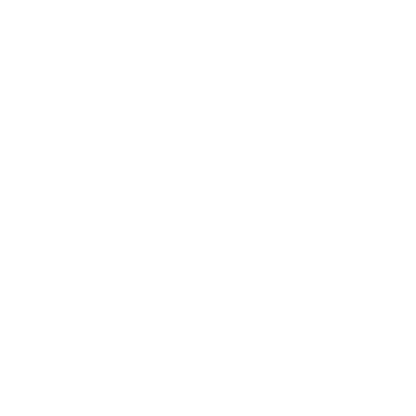

Asthma, allergy and autoimmune diseases such as diabetes, Crohn's disease, ulcerative colitis, psoriasis, rheumatoid arthritis and systemic lupus erythematosus are some of the most common chronic diseases affecting people around the world^1^,^2^,^3^,^4^,^5^,^6^,^7^,^8^. Strong evidence of heritability from twin studies has prompted large-scale genome-wide association studies (GWAS) to pinpoint the genetic risk factors that drive the pathogenesis of these complex diseases^9^. Several thousand common single-nucleotide polymorphisms (SNPs) associated with disease susceptibility have been identified; however, the vast majority of these SNPs are located in non-coding regions of the genome, and thus it has been challenging to define how these SNPs are related to the disease^10^. Moreover, the cell type(s) where disease risk-associated SNPs have the most prominent effects are unknown, thus hampering functional studies required to successfully translate GWAS discoveries to improvements in disease management. The vast array of immune and structural cell types involved in disease pathogenesis further compound this problem.

Based on recent papers from the ENCODE Project Consortium^11^,^12^ and our own analyses^13^, there is now overwhelming evidence that many disease-associated genetic variants can perturb the functions of *cis*-regulatory DNA that controls the expression of one or more neighbouring genes on the same allele, thereby influencing gene expression and disease outcome. In many cases these *cis*-regulatory regions are cell type-specific^11^, such that a region and its disease-associated genetic variants influence gene expression in just a subset of all possible cell types in the body, and therefore, these susceptible cell types are likely to be major drivers of the genetic risk for that specific disease. Thus, identifying the cell type in which *cis*-regulatory regions bearing disease-associated SNPs are selectively active may indicate the precise cell types that initiate or maintain disease pathogenesis. For example, by overlapping enhancers of naive and memory CD4^+^ T-cell subsets with all known asthma-risk SNPs, we recently showed strong enrichment of these SNPs in enhancers of memory CD4^+^ T cells that produce type 2 cytokines (T_H_2 cells), implying an important role for T_H_2 cells in asthma pathogenesis^14^.

In this study, we take a similar unbiased approach to determine the cell types that are most susceptible to the effects of SNPs located in the 17q12-q21 genetic risk locus. Originally the 17q21 locus was strongly linked to asthma susceptibility, which was confirmed by multiple other GWAS studies in diverse ethnic populations^15^,^16^,^17^,^18^,^19^,^20^ and in a large study of individuals with severe asthma^21^. Subsequently, various other autoimmune diseases such as type 1 diabetes, Crohn's disease, ulcerative colitis, psoriasis, rheumatoid arthritis, systemic lupus erythematosus and primary biliary cirrhosis were all found to share the same 17q21 risk locus, suggesting its broader implication in several diseases^2^,^3^,^4^,^5^,^6^,^7^,^8^,^22^; however, an important distinction is that the risk alleles for asthma paradoxically have a protective effect for the autoimmune diseases studied^2^,^3^,^4^,^5^,^6^,^22^. Despite the robustness of these associations with asthma and other autoimmune diseases, the molecular mechanisms by which 17q21 SNPs perturb gene expression and/or predispose carriers to disease are unclear.

The 17q21 locus harbours a dense haploblock of 136 SNPs in tight linkage disequilibrium that overlap six gene loci: *IKZF3*, *ZPBP2*, *GSDMB, ORMDL3*, *LRRC3C* and *GSDMA*^1^,^23^, of which the expression of two genes (*ORMDL3* and *GSDMB*) has been shown to be modestly affected by the asthma-risk SNPs in the locus^24^,^25^,^26^. A recent study that linked genotype to phenotype showed that children carrying the 17q21 asthma-risk SNP (rs7216389) were at higher risk of developing wheeze following rhinovirus infection (common cold), an event that is an independent predictor of asthma development later in life^26^. Although *ORMDL3* expression is elevated in children with asthma as well as in individuals with the 17q21 risk-haploblock^1^,^23^,^25^,^27^, the biological functions of ORMDL3 that are relevant to asthma pathogenesis are also poorly defined.

Here we systematically examine in which cell types the 17q21 risk haploblock has the most effect on *ORMDL3* expression, how that effect is mediated by disruption of *cis*-regulatory elements, and how cytokine production is thereby affected in T cells. We show that *ORMDL3* expression is most perturbed by the 17q21 asthma-risk SNPs in primary T cell subsets and B cells. Assessing H3K27ac (chromatin mark of active enhancers) enrichment levels in the 17q21 locus revealed an active enhancer in the first intron of *ORMDL3* that displayed genotype-dependent changes in activity. Further, we discovered that an asthma-risk SNP in this enhancer favours the binding of CTCF (rs4065275), while in contrast another linked SNP downstream of this site prevented CTCF binding (rs12936231), leading to alteration of CTCF-binding patterns in the locus. The three-dimensional (3D) organization of the 17q21 locus in CD4^+^ T cells was also modified in the asthma-risk alleles to favour recruitment of distal *cis*-regulatory elements to the *ORMDL3* promoter region. Knocking down transcript levels of *ORMDL3* in memory CD4^+^ T cells significantly increased the production of interleukin (IL)-2 following T-cell receptor stimulation.

## Results

### 17q21 asthma-risk SNPs locus overlap immune cell enhancers

**Figure 1:**
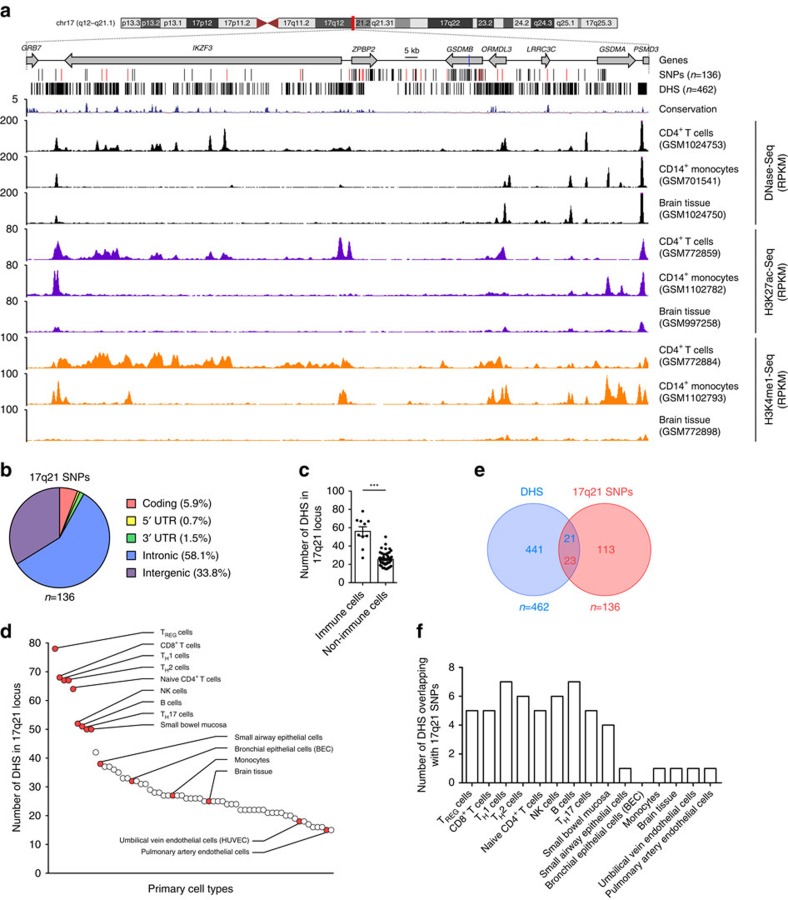
17q21 SNPs overlap immune cell enhancers. (**a**) University of California Santa Cruz (UCSC) tracks showing chromosomal location and genes present in the 17q21 locus, containing a large haplotype block of asthma-associated SNPs; the location of asthma-risk SNP rs7216389 is indicated as blue line in the gene track. Black lines indicate SNPs' genomic location, and red lines are SNPs that overlap peaks of DNase hypersensitivity sites (DHS) from multiple cell types obtained from the ENCODE Encyclopedia (version 2) provided by the ENCODE Project Consortium (see Methods). Exemplary DHS tracks, H3K27ac and H3K4me1 enrichment tracks from CD4^+^ T cells, CD14^+^ monocytes and brain tissue (from ENCODE Project and NIH Epigenomics Roadmap Consortiums) are shown along with UCSC multispecies conservation tracks. (**b**) Distribution of asthma-associated 17q21 SNPs in different genomic regions. (**c**) The average number of DHS in the 17q21 locus of immune versus non-immune cell types (*n*=10 and *n*=52, respectively) and (**d**) the 62 primary cell types (indicated as dots, profiled by the ENCODE Project Consortium, see Methods) ordered based on the number of DHS in the 17q21 locus. The top hits and discussed cell types are named and marked in red. (**e**) Overlap of DHS and 17q21 SNPs. (**f**) Number of DHS that directly overlap 17q21 SNPs in various cell types (full list in Supplementary Data set 2b). Error bars are mean±s.e.m.; ***P<0.001 by Student's unpaired two-tailed *t*-test, and following Bonferroni correction for multiple testing.

The majority of SNPs (94%, 128/136) present in the 17q21 asthma-risk haploblock are located in intronic or intergenic non-coding sequences (non-coding SNPs, ncSNPs) whose cell-specific *cis*-regulatory potential has not been fully characterized (Fig. 1a,b); as a result, the specific cell type that is most affected by the 17q21 asthma-risk variants is largely unknown. This is especially important for investigating the genetic basis of diseases such as asthma where several immune and structural cells present in the lungs have been implicated in disease pathogenesis^28^,^29^,^30^. Here we utilized the comprehensive DNase I hypersensitivity sites (DHS) data sets, generated by the ENCODE Project^31^ and NIH Epigenomics Roadmap Consortiums^32^, to first define the cell types where the 17q21 locus is selectively more active, that is, harbour a significantly higher number of *cis*-regulatory elements (DHS), as these cell types are more likely to be affected by the ncSNPs present in the locus. A total of 462 unique DHS were present in the 17q21 locus (Fig. 1a) and immune cell types had significantly more DHS when compared with non-immune cell types (*P*<0.001 by Student's unpaired two-tailed *t*-test, and following Bonferroni correction for multiple testing, Fig. 1c and see Methods); this enrichment of immune cell DHS was not observed in many of the other asthma-risk haploblocks identified by GWAS studies (Supplementary Fig. 1a and Supplementary Data set 2), suggesting that this effect in immune cells is not generic for all GWAS asthma-risk haploblocks. By ordering 62 primary cell types based on the number of DHS present, we observed that lymphocytes (T cell subsets, B cells and natural killer (NK) cells) ranked in the top 10, harbouring well over 50 DHS (Fig. 1d), whereas monocytes, lung structural cells including bronchial epithelial cells (BECs), fibroblasts and endothelial cells, and other irrelevant tissue types (for example, brain) harboured relatively fewer DHS. Among fetal tissues, the greatest number of DHS was observed in thymus and spleen (organs rich in immune cells) instead of lung tissue (the target organ affected in asthma) (Supplementary Fig. 1b). Altogether, these data indicate that the locus is highly active in several immune cell types, and that several 17q21 *cis*-regulatory regions are highly immune cell-specific (Fig. 1a,c,d).

About 17% (23/136) of the SNPs in the 17q21 asthma-risk haploblock directly overlap DHS in the locus (Fig. 1e). Importantly, more than five of the DHS present in immune cells, such as T-cell subsets, B cells and natural killer cells, directly overlap asthma-risk SNPs, whereas DHS present in monocytes, epithelial cells or other irrelevant tissue (brain) overlap one or no asthma-risk SNPs (Fig. 1f). Thus, our *in silico* analysis of enhancer profiles suggests that expression of 17q21 genes is most likely to be perturbed in immune cell types.

### *ORMDL3* expression is most affected in primary T cells

**Figure 2:**
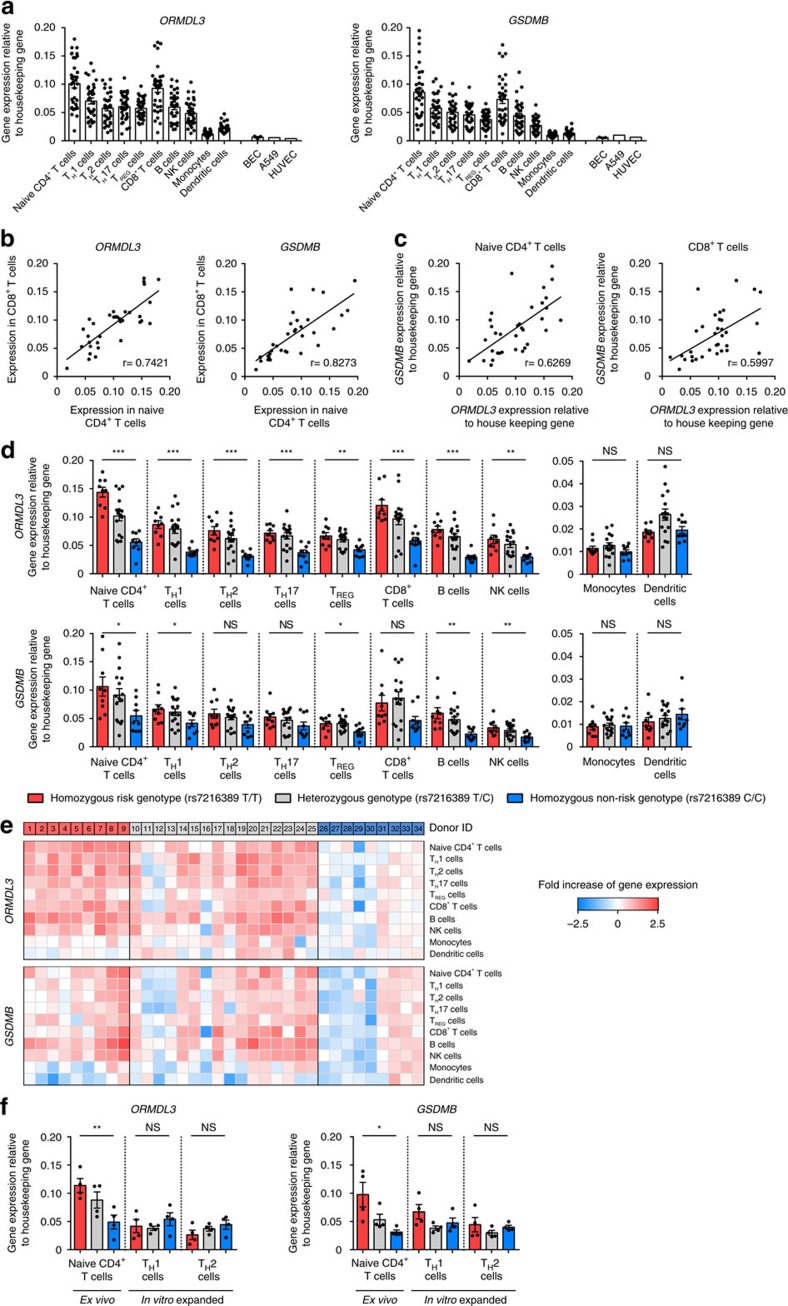
17q21 SNPs have pronounced effects on *ORMDL3* expression in primary T and B cells. (**a**) Real-time PCR quantification of *ORMDL3* and *GSDMB* transcript levels (relative to the housekeeping gene *YWHAZ*) in the indicated primary immune cell types (*n*=34 donors), bronchial epithelial cells (BEC; *n*=3), lung cancer cells (A549; *n*=1) and human umbilical vein endothelial cells (HUVEC; *n*=1). (**b**) Correlation of *ORMDL3* and *GSDMB* transcript levels between naive CD4^+^ T cells and CD8^+^ T cells. (**c**) Correlation between *ORMDL3* and *GSDMB* transcript levels in naive CD4^+^ T cells and CD8^+^ T cells. (**d**) *ORMDL3* and *GSDMB* transcript levels in the indicated cell types from donors categorized based on the genotype for asthma-associated SNP rs7216389 (homozygous risk (T/T): *n*=9, red bar; heterozygous (T/C): *n*=16, grey bar; homozygous non-risk (C/C): *n*=9, blue bar). (**e**) Heat maps show Log2 fold change in transcript levels of *ORMDL3* and *GSDMB* relative to the average expression levels seen in the corresponding cell types from donors with the non-risk (C/C) genotype; the donors are ordered based on genotype and the number indicates the unique ID (Supplementary Data set 3). (**f**) *ORMDL3* and *GSDMB* transcript levels in naive CD4^+^ T cells and in *in vitro* expanded and polarized T_H_1 or T_H_2 cells from matched donors (see Methods), categorized based on the genotype as in **d**. Each dot represents data from a single donor. Error bars are mean±s.e.m.; *P<0.05, **P<0.01, and ***P<0.001 by Student's unpaired two-tailed *t*-test; NS, not significant; *r* value indicates the Spearman correlation coefficient.

In 10 primary immune cell types freshly isolated from peripheral blood of 34 subjects enrolled in the La Jolla Institute for Allergy and Immunology's (LJI) Normal Blood Donor Program (Supplementary Fig. 2), we assessed expression of two genes in the 17q21 locus (*ORMDL3* and *GSDMB*) whose expression levels have been shown to be affected by 17q21 SNPs in previous studies^24^,^25^,^26^. Compared with non-immune cells, such as BECs, lung cancer cell lines (A549), and human umbilical vein endothelial cells, both genes were expressed at relatively high levels in most primary immune cell types with the exception of monocytes and dendritic cells (Fig. 2a). Among the 34 subjects, expression of *ORMDL3* and *GSDMB* genes were both correlated between the cell types of higher transcriptional activity (Spearman correlation value of 0.74 and 0.83, respectively, between naive CD4^+^ T cells and CD8^+^ T cells, Fig. 2b and Supplementary Fig. 3a). Though the expression levels of *ORMDL3* and *GSDMB* were variable across donors (Fig. 2a,b), the two were positively correlated across the 34 donors (Spearman correlation value of 0.63 in naive CD4^+^ T cells, Fig. 2c), suggesting a potentially co-regulated expression of these two genes in several cell types (Supplementary Fig. 3b).

Based on genotype at rs7216389 (see Supplementary Data set 3)^1^, located in the gene body of *GSDMB*, subjects were classified into three groups: (i) homozygous for the asthma-risk allele (TT genotype, *n*=9), (ii) homozygous for the non-risk allele (CC genotype, *n*=9) and (iii) carrying heterozygous alleles (TC genotype, *n*=16). Previous studies have reported a modest increase (∼1.3 to 1.8-fold in peripheral blood mononuclear cells (PBMCs) and lymphoblastoid cell lines)^1^,^24^,^26^,^27^ in the expression of *ORMDL3* and *GSDMB* transcripts in subjects carrying the asthma-risk alleles. In comparison, we found a much greater increase in purified primary immune cell types, with some cell types such as naive CD4^+^ T cells and B cells displaying a nearly threefold increase in *ORMDL3* expression (Fig. 2d, left panel and Fig. 2e). In sharp contrast, monocytes and dendritic cells showed no genotype-dependent effect on gene expression (Fig. 2d, right panel), implying that the effect of the 17q21 asthma-risk variants is cell-specific and restricted to some primary immune cell types. These findings concur with our prediction based on overlap of 17q21 asthma-risk variants with cell-specific *cis*-regulatory elements (Fig. 1f).

We noted that the effect of asthma-risk variants on *ORMDL3* expression was completely lost when primary naive CD4^+^ T cells were expanded in culture for a few days (Fig. 2f), suggesting *in vitro* expansion *per se* may dampen the effects of the 17q21 variants. Recent studies have shown that 17q21 risk variants have only modest effects on *ORMDL3* expression in *in vitro* expanded human lymphoblastoid cell lines^1^,^27^, and in the case of primary BECs these variants exert no effect^33^. Such divergent effects of the asthma-risk variants among cell types indicate that the upstream pathway positively regulating the *ORMDL3* locus is sensitive to the effects of genetic variants only in certain cell types; therefore, the functional consequences of the underlying genetic risk are likely to be best observed in the cell types most sensitive to the effects of asthma-risk variants, in this case T cell subsets. Since CD4^+^ T cells have a central role in orchestrating asthma pathogenesis^34^, we focused our functional studies on this subset of T cells.

### 17q21 SNPs affect function of *ORMDL3* intronic enhancer

**Figure 3:**
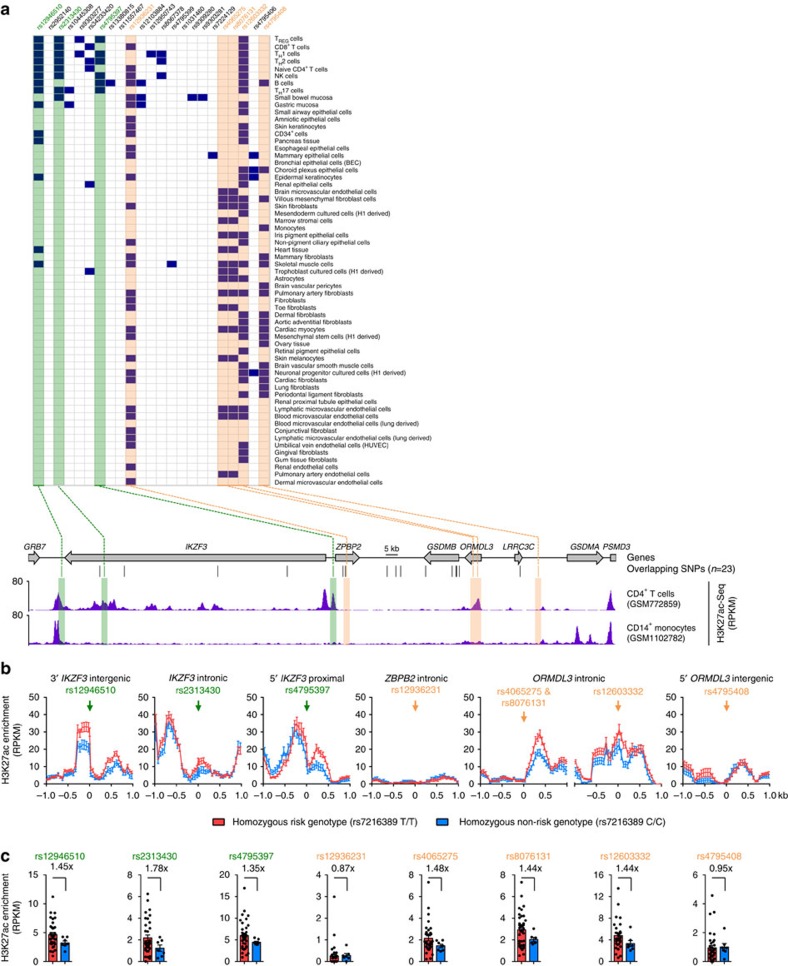
17q21 SNPs affect the function of an intronic enhancer in *ORMDL3*. (**a**) 17q21 SNPs (columns) that overlap with DHS (shown in dark blue squares) in different primary cell types (rows), as described in Fig. 1e,f. Green boxes highlight SNPs that overlap DHS enriched in lymphocytes and orange boxes highlight SNPs that overlap DHS in many immune and non-immune cell types. Bottom panel shows UCSC tracks of the 17q21 locus, along with asthma-associated SNPs that overlap DHS. H3K27ac enrichment tracks of CD4^+^ T cells and CD14^+^ monocytes (from NIH Epigenomics Roadmap) are shown below. Boxes highlight SNPs of interest that overlap DHS. (**b**) Tracks showing comparison of average H3K27ac enrichment values (RPKM) (in each 50-bp window spanning 1 kb region on either side of the SNPs of interest) between homozygous risk (rs7216389 T/T, *n*=36) and non-risk (rs7216389 C/C, *n*=7) samples. Arrow indicates location of the SNP; error bars are mean±s.e.m. (**c**) H3K27ac enrichment values for a 200-bp region around the indicated asthma-risk SNP. Each dot represents data from a single assay; error bars indicate mean±s.e.m.; numbers indicate average fold change in H3K27ac enrichment between carries with risk and non-risk allele.

We next wanted to predict the *cis*-regulatory elements (enhancers) that are affected by asthma-risk variants present in the 17q21 locus. Among the 23 SNPs that directly overlap 21 DHS in the 17q21 locus (shown in Fig. 1e), we focused our analysis on two groups of SNPs: (i) those that overlap *cis*-regulatory elements (DHS) selectively enriched in immune cell types where *ORMDL3* expression is affected (Fig. 3a, *green highlighted boxes*), and (ii) those that overlap DHS present in immune cell types as well as a wide range of other primary cell types (Fig. 3a, *orange highlighted boxes*). The latter SNPs could impact the function of multiple other cell types potentially involved in disease pathogenesis.

Three asthma-risk SNPs (rs4065275, rs8076131 and rs12603332) overlapped DHS in the first intron of *ORMDL3* (Fig. 3a, top panel); this ∼3.0 kb intronic region was also highly enriched for the H3K27ac mark, suggestive of active enhancer activity, in CD4^+^ T cells but not monocytes (Fig. 3a, bottom panel). Next, we asked if the activity of this *ORDML3* intronic enhancer, as judged by enrichment levels of H3K27ac, was modified by the asthma-risk alleles. We utilized a large data set of H3K27ac ChIP-Seq assays in memory CD4^+^ T cells to assess effects of asthma-risk genotype on enhancer activity. An increase (∼1.44 fold) in H3K27ac enrichment was observed in subjects carrying the asthma-risk allele when compared with those carrying the non-risk allele (Fig. 3b), suggesting that the activity of this enhancer is altered by asthma-risk SNPs.

In addition to the *ORMDL3* intronic enhancer, two other DHS regions that overlap asthma-risk SNPs were also present in 25–60% of the cell types analysed, that is were not strictly cell-type specific (Fig. 3a, top panel). These regions included the *ZPBP2* intronic region that overlaps rs12936231 and the intergenic region that is 25 kb downstream of *ORMDL3* intergenic region overlapping ncSNP (rs4795408).

The three DHS sites that were selectively enriched in lymphocytes were present near the *IKZF3* gene, some located well over 100 kb away from the *ORMDL3* promoter. The distal *IKZF3* intergenic region overlaps ncSNP (rs12946510) and similar to the *ORMDL3* intronic enhancer, this region showed increased activity (H3K27ac enrichment) in subjects carrying the asthma-risk allele (Fig. 3b,c). The other two regions were (5′) proximal and intronic *IKZF3* regions, which overlap the ncSNPs rs2313430 and rs4795397, respectively (Fig. 3b,c). Overall, our observations point to several potentially important enhancer elements in the 17q21 locus, especially the *ORMDL3* intronic enhancer, whose function is affected by asthma-risk variants.

### 17q21 asthma-risk SNPs switch CTCF-binding sites

**Figure 4:**
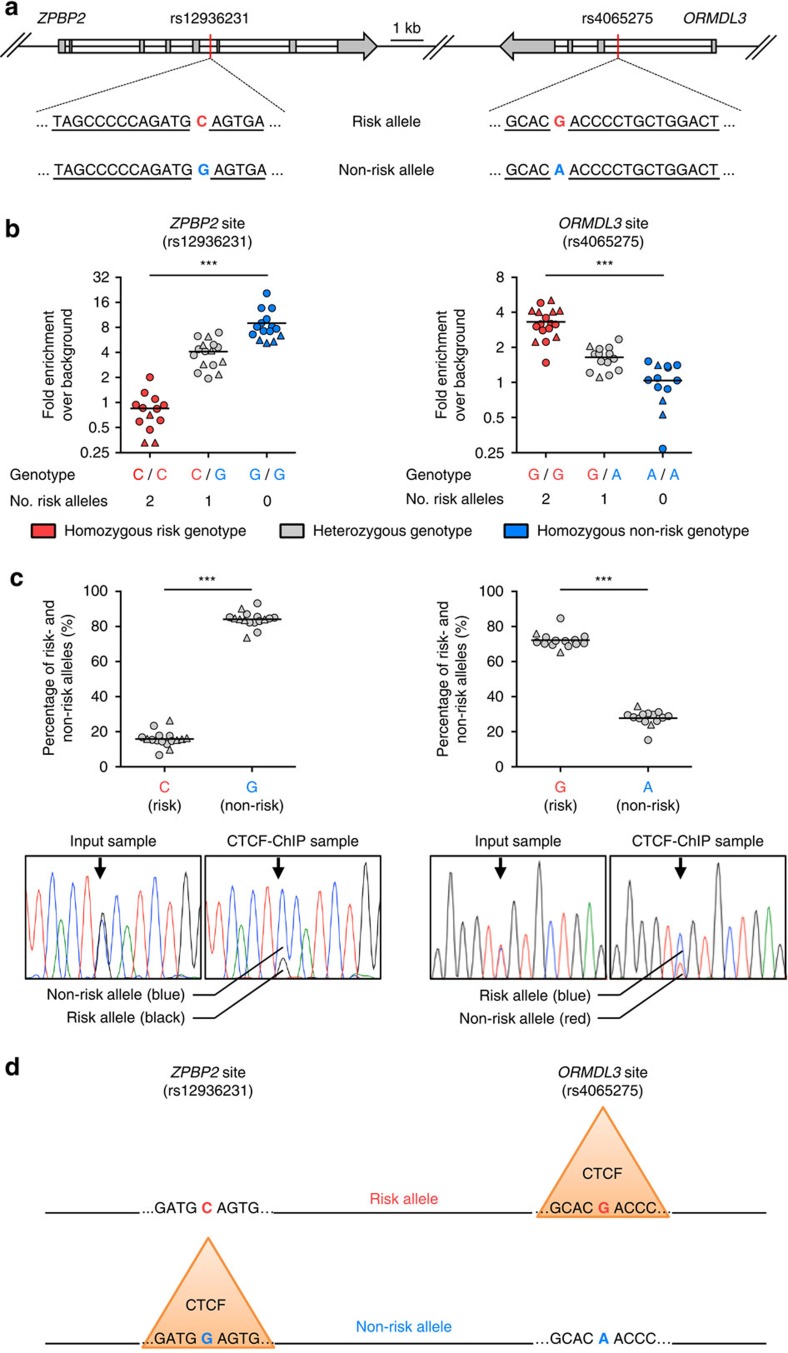
CTCF-binding sites are switched by 17q21 SNPs. (**a**) Schematic representation of the 17q21 locus containing CTCF-binding motifs (underlined sequence) that overlap with the linked SNPs rs12936231 (C/G; *ZPBP2* site) and rs4065275 (G/A; *ORMDL3* site); the asthma-risk SNP is indicated in red, the non-risk SNP in blue. (**b**) Real-time PCR quantification of DNA sequences, containing the 17q21 linked SNPs rs12936231 (*ZPBP2* site) or rs4065275 (*ORMDL3* site), after anti-CTCF ChIP of chromatin extracts obtained from polarized T_H_1, T_H_2 cells (shown as circles) and primary CD8^+^ T cells (shown as triangles; see Methods) of donors categorized based on allelic status of SNP overlapping the respective CTCF motif (as shown in **a**). Data are expressed as fold enrichment relative to an irrelevant background control; data were obtained from four independent experiments and each dot represents data from a single ChIP assay; *n*=44 assays from 15 subjects (see Methods and Supplementary Fig. 4c,d). (**c**) Percentage of DNA sequences containing the risk and non-risk SNPs at rs12936231 (*ZPBP2* site) or rs4065275 (*ORMDL3* site) following Sanger sequencing of DNA obtained following anti-CTCF ChIP assay performed in heterozygous donors shown in **b** (see Methods and Supplementary Fig. 4b); shown below are nucleotide traces from Sanger sequencing of ChIP and input DNA from a representative experiment. (**d**) Schematic representation of the switch in CTCF binding introduced by the linked asthma-risk SNPs (rs12936231 and rs4065275) in the 17q21 locus; orange triangles represent binding of CTCF to the preferred allele. ***P<0.001 by Mann–Whitney *U*-test.

We performed an *in silico* analysis to identify the transcription factors whose binding to the *cis*-regulatory regions described above is perturbed by asthma-risk SNPs. Our evaluation pointed to perturbation of two CTCF-binding motifs as well as motifs of several other transcription factors such as IRF1, PAX4, TCF3 (Fig. 4a and Supplementary Data set 5). We focused on CTCF binding because it directly overlaps the *ORMDL3* intronic enhancer region (Fig. 4a), and CTFC protein has been shown to play an important role in enhancer–promoter interactions and as an insulator protein^35^. The first CTCF-binding motif was altered by an asthma-risk SNP (rs4065275) in the *ORMDL3* intronic region causing an A-to-G change in the motif, which is predicted to enhance the binding of CTCF (Fig. 4a and Supplementary Data set 5). We experimentally confirmed that CTCF binding in the *ORMDL3* intronic region was significantly enriched in both expanded and primary T cells from subjects carrying the risk allele (Fig. 4b). As a control region, we selected a well-known invariant CTCF binding site ∼50 kb proximal to *ORMDL3* (near the *GSDMA* gene), which did not harbour any motif-disrupting asthma-risk SNP; this region did not show any significant change in CTCF binding (Supplementary Fig. 4a). In heterozygous subjects, we performed allele-specific analysis of CTCF-bound DNA and found strong preference for the risk allele (Fig. 4c and Supplementary Fig. 4b). Thus, we confirmed that the presence of asthma-risk SNP (rs4065275) introduces a CTCF-binding site in the *ORMDL3* intronic region that harbours an active enhancer.

The second CTCF-binding motif was altered by asthma-risk SNP (rs12936231) in the *ZPBP2* intronic region causing a G-to-C change in the CTCF motif, which was predicted in our *in silico* analysis and previously shown to impair CTCF binding^27^. We confirmed in CD4^+^ and CD8^+^ T cells that CTCF binding was almost completely lost in the *ZPBP2* intronic region from subjects carrying the asthma-risk SNP, and this finding was true at the allele-specific level (Fig. 4b,c). Thus, the asthma-risk haploblock harbours linked SNPs that switch the binding site of CTCF from the *ZPBP2* to the *ORMDL3* intronic region (Fig. 4d).

### 17q21 SNPs modify *ORMDL3* promoter–enhancer interactions

**Figure 5:**
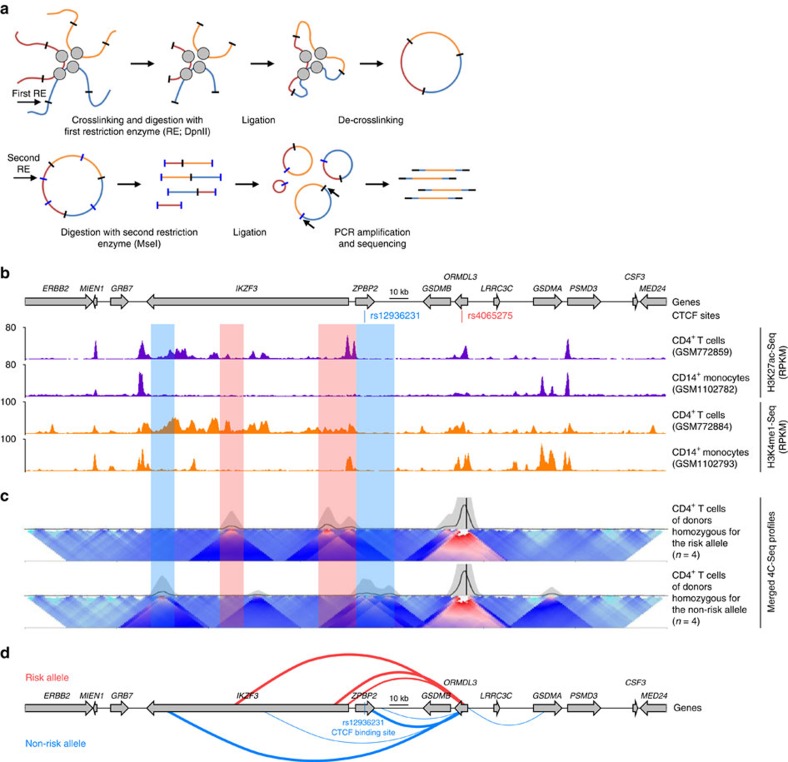
Asthma-risk SNPs modify long-range *ORMDL3* promoter-enhancer interactions. (**a**) Schematic representation of the 4C-Seq assay and the choice of restriction enzymes used for various steps. (**b**) UCSC gene tracks of 17q21 locus (chr17: 37,849,238 - 38,189,238 (hg19); 340 kb) showing the CTCF-binding sites that overlap with the linked SNPs rs12936231 (C/G; *ZPBP2* site) and rs4065275 (G/A; *ORMDL3* site), along with H3K27ac and H3K4me1 enrichment tracks of CD4^+^ T cells and CD14^+^ monocytes. (**c**) Merged 4C-Seq domainograms, using colour-coded intensity values to indicate relative levels of interactions (red denotes the strongest interactions and dark blue to turquoise representing gradually decreasing frequencies), generated using 4Cseqpipe^40^ (see Methods) are displayed for CD4^+^ T cells from subjects homozygous for the risk (*n*=4) and non-risk alleles (*n*=4); the bait region (*ORMDL3* promoter) is marked as a black line. Shaded boxes highlight regions that interact with the bait region at the *ORMDL3* promoter in the risk and non-risk alleles (shown in red and blue colour, respectively). (**d**) Schematic representation of the DNA regions interacting with the *ORMDL3* promoter in the asthma-risk (red colour) and non-risk alleles (blue colour), the CTCF-binding site in the *ZPBP2* region of the non-risk alleles in shown as a blue line.

Since CTCF has a major role in long-range interactions of *cis*-regulatory elements to cognate promoter regions^36^,^37^,^38^,^39^, we hypothesized that the switching of CTCF-binding sites may affect the 3D architecture (organization) of the 17q21 locus to favour enhanced transcription of *ORMDL3* in the asthma-risk alleles. In order to define all the distal *cis*-regulatory elements that potentially regulate *ORMDL3* expression in an asthma-risk genotype-dependent manner, we performed 4C-Seq assays (see Methods) in primary CD4^+^ T cells from subjects homozygous for the risk (*n*=4) and non-risk alleles (*n*=4). By designating the *ORMDL3* promoter as the bait for 4C, we detect at a high resolution the physical interactions of any potential *cis*-regulatory element in the extended 17q21 locus with the *ORMDL3* promoter region (Fig. 5a,b). The 4C-Seq assay^40^,^41^ conditions were initially optimized in a human T cell line (HUT-78) to robustly detect long-range interactions of the *ORMDL3* promoter (Supplementary Fig. 5a and see Methods).

Genomic regions interacting with the *ORMDL3* promoter region were determined using two complementary analysis methods for 4C data (4C-ker^42^ and 4Cseqpipe^40^, see Methods). We first pooled data from all risk and non-risk subjects and identified several interacting regions including the CTCF-bound regions near *ZBPB2* and *ORMDL3* gene loci, the *IKZF3* promoter region and a number of other potential enhancer regions in the *IKZF3* intronic region (Fig. 5c), suggesting extensive long-range interactions of the *ORMDL3* promoter region in primary human CD4^+^ T cells. Most strikingly, the *IKZF3* promoter region and other potential enhancers in the *IKZF3* intronic region that were enriched for H3K27ac and H3K4me1 marks in CD4^+^ T cells but not monocytes (that is, selectively active in CD4^+^ T cells) appeared to interact with the *ORMDL3* promoter region exclusively in subjects carrying the 17q21 asthma-risk allele (Fig. 5c, *red highlighted boxes;* for example, region chr17: 38,022,212-38,022,898 (hg19) with an adjusted *P* value of 0.000736 using the 4C-ker method^42^, Supplementary Data set 6), whereas the *ORMDL3* promoter region of non-risk alleles interacted with the CTCF-binding site in the *ZPBP2* intronic region (Fig. 5c, *blue highlighted boxes*), which presumably insulated (blocked) the interactions to the adjacent active *IKZF3 cis*-regulatory elements. Though subjects in each group showed some variability, potentially due to their genotypic variation within this locus as we classified subjects as risk and non-risk only based on genotype at rs7216389, rs4065275 and rs12936231 (see Methods), the overall pattern was consistent across most donors (Supplementary Fig. 5b and Supplementary Data sets 6 and 7).

Our results suggest that the 3D organization of the 17q21 locus is modified in memory CD4^+^ T cells from subjects with the 17q21 asthma-risk alleles to favour selective recruitment of the *IKZF3 cis-*regulatory elements to the *ORMDL3* promoter region (Fig. 5d), which correlated with its increased transcriptional activity.

### ORMDL3 negatively regulates IL-2 production by CD4^+^ T cells

**Figure 6:**
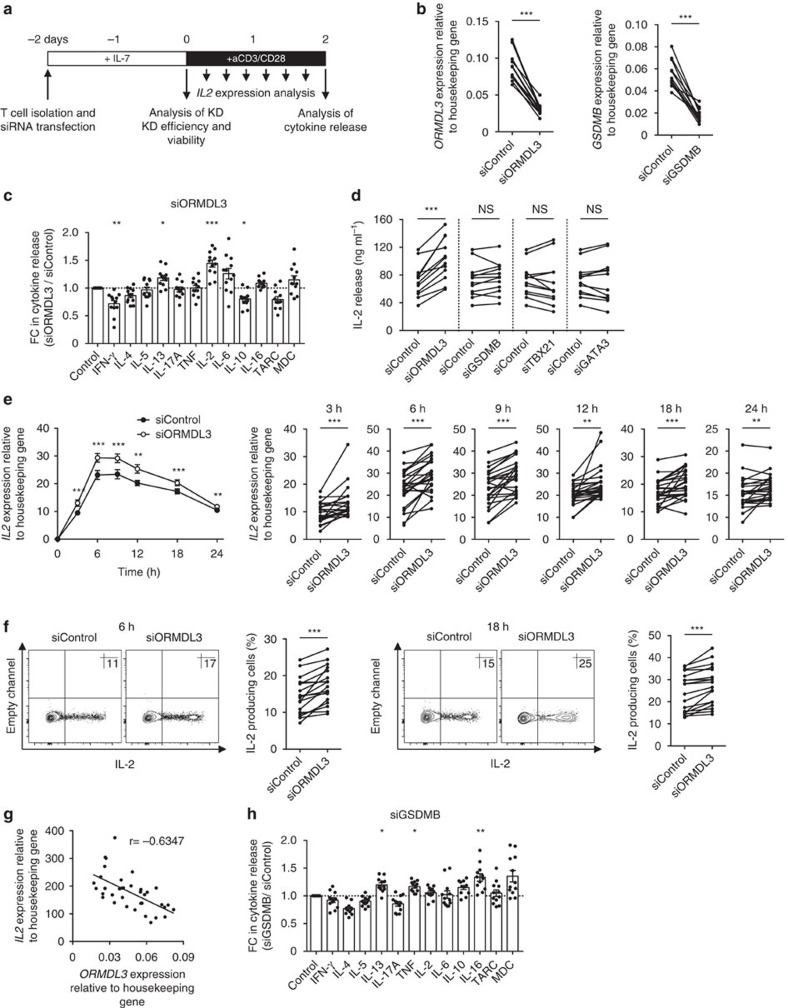
ORMDL3 negatively regulates IL-2 production by CD4^+^ T cells. (**a**) Experimental design used for assessing effects of knocking down genes of interest in memory CD4^+^ T cells. (**b**) Real-time PCR quantification of *ORMDL3* and *GSDMB* transcript levels (relative to the housekeeping gene *YWHAZ*) in memory CD4^+^ T cells 48 h after knock down with control siRNA pools, *ORMDL3* or *GSDMB* siRNA pools (*n*=12 donors). (**c**) Effects of *ORMDL3* knockdown on cytokine release by memory CD4^+^ T cells activated for 48 h with antibodies to CD3 and CD28; data are expressed as fold change (FC) relative to control siRNA-treated conditions; error bars indicate mean±s.e.m. (**d**) Absolute values of IL-2 protein levels in culture supernatants from cells treated with the indicated siRNA pools. Data are presented as means of biological duplicates. (**e**) Time course of *IL2* mRNA expression in activated memory CD4^+^ T cells (*n*=24) following treatment with *ORMDL3* (open circles) or control siRNA pools (closed circle); data from each donor for different time points after stimulation is shown in the right panel. (**f**) Representative FACS plots showing intracellular staining of IL-2 in memory CD4^+^ T cells activated for 6 or 18 h (after knockdown with siRNA pool for *ORMDL3* or control siRNA); percentage of IL-2 producing cells in each donor is shown to the right (*n*=18). (**g**) Correlation of the levels of *ORMDL3* transcripts (measured at baseline) and *IL2* transcripts produced following *ex vivo* stimulation of memory CD4^+^ T cells (*n*=36) with phorbol myristate acetate (PMA) and Ionomycin for 4 h. (**h**) Effects of *GSDMB* knockdown on cytokine release by memory CD4^+^ T cells, as described in **c**. Each dot represents data from a single donor. *r*, Spearman correlation coefficient; *P<0.05, **P<0.01, and ***P<0.001 by Student's paired two-tailed *t*-test, and following Bonferroni correction for multiple testing in **c**,**d** and **h** (see Methods).

Next, we wanted to determine the functional consequences of altered levels of *ORMDL3* transcripts in primary CD4^+^ T cells, as 17q21 SNPs had the greatest impact on *ORMDL3* expression in this cell type. ORMDL3 is an endoplasmic reticulum membrane protein, and studies in cell lines and in epithelial cells have indicated its involvement in Ca^2+^ signalling, sphingolipid metabolism and unfolded-protein responses^43^,^44^,^45^; however, its function in primary T cells has not been fully investigated. Here we sought to determine the functional role of ORMDL3 in memory CD4^+^ T cells with a specific focus on cytokines released following activation. Freshly isolated human memory CD4^+^ T cells were transfected with small interfering RNA (siRNA) pools targeting the *ORMDL3* transcript, cultured for 48 h to achieve adequate gene knockdown, and then activated by T cell receptor ligation (anti-CD3) and co-stimulation (anti-CD28) to study effects on cytokine production (Fig. 6a and see Methods).

We achieved over 50% reduction in *ORMDL3* transcript levels without affecting cell viability due to transfection (Fig. 6b and Supplementary Fig. 6a,c). Among the cytokines and chemokines measured, the strongest effect (>40% change) was seen for IL-2 production, which was significantly increased in the *ORMDL3* siRNA-treated conditions compared to control siRNA conditions (Fig. 6c). IL-2 production was not affected by knocking down *GSDMB* or other control genes such as *TBX21* and *GATA3* (Fig. 6d).

At the transcriptional level, we observed similar effects on activation-induced expression of *IL2* transcripts following *ORMDL3* knockdown (Fig. 6e). Notably, we observed significant effects at very early (3, 6 h) and late (18 h) time points following stimulation, suggesting sustained effects of *ORMDL3* knockdown (Fig. 6e, right panel). We also confirmed these effects at the protein level using fluorescence-activated cell sorting (FACS)-based intracellular cytokine detection assay (Fig. 6f). We next asked if the negative regulatory effect on *IL2* transcription was also observed in unmanipulated T cells from subjects with varying levels of *ORMDL3* expression. As expected, we found a striking correlation between baseline *ORMDL3* expression and the levels of *IL2* transcripts produced following brief *ex vivo* stimulation of memory CD4^+^ T cells with phorbol myristate acetate (PMA) and Ionomycin (Fig. 6g), suggesting that this correlation may also hold *in vivo*. Altogether, our results provide strong evidence that ORMDL3 negatively regulates IL-2 production in CD4^+^ T cells, and that overexpression of *ORMDL3* in subjects carrying the asthma-risk alleles is likely to reduce IL-2 production *in vivo*, which in turn may have important functional effects that contribute to the development of asthma^46^.

*ORMDL3* knockdown also led to modest changes in the production of other cytokines such as interferon-γ (IFN-γ), IL-10 and IL-13, suggesting that ORMDL3 is likely to have a broader spectrum of functions in T cells. Knocking down *GSDMB* led to modest changes in the production of TNF, IL-13 and IL-16 (Fig. 6h), suggesting that the product of this gene is also likely to affect the function of T cells. Overall, our results suggest that physiological alteration in the levels of *ORMDL3* and *GSMDB* induced by the asthma-risk allele may be sufficient to modulate the functional capacity of T cells.

## Discussion

The overwhelming majority of the sequence variants (SNPs) associated with disease-risk haplotypes in GWAS studies do not change protein coding, implying that a proportion of them may be located in regulatory regions which act *in cis* to alter gene expression^1^,^16^,^20^,^47^. Since in many instances *cis*-regulatory regions are highly cell type-specific, the effects of SNPs that affect their function are also likely to be more pronounced in just a few cell types. The failure to link non-coding risk SNPs to the cell types in which they affect function has significantly hampered the progress of functional studies aimed at identifying biological effects of GWAS SNPs. In this study, we attempted to solve this problem by taking a comprehensive approach to predict and test the effects of 17q21 asthma-risk SNPs on expression of *ORMDL3* in various cell types. We show that primary immune cell types, with the exception of monocytes and dendritic cells, but not lung structural cells such as BECs, are most sensitive to the effects of 17q21 SNPs. Notably, this approach allowed us to narrow down the cell types in which ORMDL3 potentially influences disease outcomes, and, therefore, perform detailed functional studies in a relevant cell type (primary human CD4^+^ T cells) and show that ORMDL3 negatively regulates the transcription of *IL2*. Given the pleotropic role of IL-2 in modulating the differentiation and function of T_H_ cell subsets, it is likely that this effect on IL-2 production could be pivotal for driving genetic risk for asthma and autoimmunity.

Previous studies have examined the function of the conserved ORM family proteins in yeast^48^,^49^ (which includes ORMDL3 in humans), as well as ORMDL3 itself in various mammalian cell lines and in primary cell types relevant to asthma pathogenesis such as epithelial cells, eosinophils, mast cells and whole PBMCs^23^,^43^,^44^,^45^,^50^,^51^,^52^,^53^,^54^. ORM family proteins were originally shown to mediate sphingolipid metabolism in yeast^48^,^49^, which was subsequently confirmed in mammalian cells^44^. As an endoplasmic reticulum membrane protein, ORMDL3 has been shown to be involved in endoplasmic reticulum-mediated Ca^2+^ homeostasis by depleting Ca2^+^ stores of the endoplasmic reticulum through an interaction with the SERCA pump in the endoplasmic reticulum^43^,^50^. In this context, ORMDL3 was shown to diminish the translocation of the nuclear factor of activated T cells (NFAT) in Jurkat cell lines and modestly reduce cytokine release after stimulation^50^. Therefore, it is likely that the negative regulatory effects of ORMDL3 on IL-2 production in primary T cells are also due to perturbation of Ca^2+^ signalling and NFAT function following T cell activation.

Overexpression of ORMDL3 in BECs has been shown to activate components of endoplasmic reticulum stress or unfolded protein response, resulting in increased expression of several pro-inflammatory molecules involved in airway remodelling and inflammation^45^,^51^. Recent studies in murine primary eosinophils and mast cells have revealed opposing functions in these cell types; in eosinophils ORMDL3 positively regulates its activation and function by influencing cell shape change, adhesion and recruitment to sites of inflammation *in vivo*, while in mast cells ORMDL3 negatively regulates activation-induced expression of pro-inflammatory mediators and chemotactic responses^51^,^53^. Thus, it appears that the function of ORMDL3 may vary considerably depending on the cell type, and, therefore, it will be important to first know whether the 17q21 asthma-risk SNPs in fact affect the expression of *ORMDL3* in these cell types; this information will allow determination of whether these cell types are the dominant factors driving the 17q21-related genetic risk of asthma.

While all SNPs in genetic linkage may be statistically significantly associated with asthma, only a subset will be functionally relevant. For instance, the 17q21 locus has a dense haploblock of 136 SNPs linked to asthma risk, a number too large to allow exploration of each SNP's potential function. In determining which SNPs are likely to influence pathophysiology, our basic assumption is that a significant fraction of ncSNPs that are deemed to be functional in asthma act on *cis*-regulatory elements that are important for controlling the expression of 17q21 genes. Therefore, to identify functional SNPs in the 17q21 haploblock, we first focused on SNPs that overlap *cis*-regulatory regions that are active in several cell types, especially those (for example, CD4^+^ T cells) in which expression of *ORMDL3* is affected by the 17q21 SNPs, and then tested whether the presence of these SNPs alters epigenetic profiles, that is, activity at relevant *cis*-regulatory DNA regions. We identified an enhancer region in the first intron of the *ORMDL3* gene that overlapped three 17q21 asthma-risk SNPs (rs4065275, rs8076131, and rs12603332), and this region showed increased activity in subjects carrying the asthma-risk allele. The rs4065275 variant introduced a binding site for the multi-functional zinc finger protein CTCF, whereas the linked rs12936231 variant, about 55 kb downstream of *ORMDL3*, disrupted a second CTCF-binding site, which together results in interchanging of two CTCF sites in the vicinity of the *ORMDL3* gene locus.

CTCF binds insulator regions in the genome^55^,^56^,^57^, where it forms gene boundaries by blocking interactions between distal enhancers and promoters; when present in introns, it influences the efficiency of pre-mRNA splicing at weak splice sites by altering the processivity of RNA polymerase II (pol II)^35^; however, we did not observe any changes in splicing of *ORMDL3* transcripts (data not shown). More recently, genome-wide analysis of CTCF-binding patterns coupled with long-range chromatin interaction studies (ChIA-PET) have shown that CTCF has a pivotal role in promoting interaction of multiple enhancers to cognate gene promoters^58^. Therefore, we hypothesized that the switching of CTCF sites by asthma-risk SNPs may alter the three-dimensional structure of chromatin in the 17q21 locus and promote the interaction of active enhancer regions with their cognate promoter (*ORMDL3*), thereby increasing *ORMDL3* expression in carriers of the asthma-risk SNPs. Experimentally, we confirmed genotype-dependent differential looping (altered 3D organization) in the 17q21 locus that resulted in the recruitment of active cell-specific *cis*-regulatory elements (for example, of the 5′ *IKZF3* proximal region which overlaps the asthma-risk SNP rs4795397) to the *ORMDL3* promoter in asthma-risk alleles. Depending on the composition of the haploblock for a given donor (combination of SNPs), the differential looping may also recruit enhancer regions that themselves are affected by SNPs and thus creating variable and donor-specific reorganization of the 17q21 locus. These mechanisms may contribute to the exquisite cell type-specific effects of 17q21 SNPs on gene expression in people with various genotypes.

The discovery of new drug targets in a human disease requires identification of the pathogenic cell type(s) as well as the molecular factors and pathways that contribute to the disease. Our current knowledge of these points is incomplete for many diseases. The approach we have taken to improve our understanding of the genetic basis of asthma and autoimmunity aims to address some of these issues and may be applied to various other disease-associated genetic loci to unravel disease-relevant cell types and molecular pathways.

## Methods

### Study subjects and sample processing

Blood samples from 34 donors were obtained from LJI's Normal Blood Donor Program after written informed consent. Ethical approval for the use of this material was obtained from the Institutional Review Board (IRB) of the LJI. Only for the data shown in Fig. 3b,c, additional samples were obtained from another cohort of 38 donors (only subjects homozygous for either the asthma risk- or non-risk variant at rs7216389), after written informed consent. The genotyping of the 17q21 SNPs rs7216389, rs12936231 and rs4065275 was performed using the automated Sanger DNA sequencing platform (GENEWIZ, Inc.) with the primers listed in Supplementary Table 1. Details of the genotype of 17q21 SNPs, age and gender of study subjects are provided in Supplementary Data set 3.

For isolating immune subtypes from peripheral blood samples, PBMCs were first separated into a CD4^+^ memory cell fraction and remaining cells by use of the Memory CD4^+^ T Cell Isolation Kit (Miltenyi Biotec). Both populations were stained with cocktails of fluorescently conjugated antibodies (see Supplementary Table 2) and sorted on a FACSAria-II (Becton Dickinson) using the gating strategy shown in Supplementary Fig. 2. Sorted cells were washed, and directly lysed in TRIzol solution (Invitrogen) for subsequent isolation of total RNA or fixed for ChIP assays as described previously^14^.

### Gene expression studies

Total RNA was extracted using the miRNeasy Micro Kit (Qiagen); complementary DNA was reverse-transcribed with the SuperScript III First-Strand Synthesis System (Life Technologies). Real-time PCR employed the Fast Start Universal SYBR Green Master Mix (Roche); see Supplementary Table 1 for primer sequences. Data were acquired on the StepOnePlus Real-Time PCR System (Applied Biosystems); all results are presented in arbitrary units relative to expression of the housekeeping gene *YWHAZ*^59^.

### Micro-scaled ChIP-Seq for H3K27ac and bioinformatics analysis

Samples from an independent cohort of 38 donors (as described above) were utilized for this analysis (Supplementary Data set 3). A total of 43 ChIP-Seq assays (including 5 technical replicates) were performed as described previously^14^,^60^. Briefly, purified chromatin from 1 × 10^5^ T_H_2 cell enriched CD4^+^ memory subset (CD3^+^CD4^+^ CD25^−^CD45RA^−^CCR4^+^) was immunoprecipitated with a polyclonal anti-H3K27ac antibody (Lot #GR184333-1; ab4729; Abcam). Chromatin was incubated with 0.5 μg antibody pre-coated to 5 μl of protein A-coated magnetic beads (Invitrogen). Immunocomplexes were captured, washed and eluted; DNA was purified and used for whole-genome amplification as described previously^14^. The samples then underwent library preparation using the Illumina TruSeq Nano DNA Library Prep Kit following the manufacturer's instructions. Libraries were sequenced on an Illumina HiSeq 2500 sequencer to obtain >10 million uniquely mapped 50-bp single-end reads.

ChIP-Seq data was processed as described^14^. To generate the H3K27ac tracks shown in Fig. 3b, sequencing coverage was calculated for the six regions (+/−1 kb around the SNP site of interest) at 50 bp windows after extending each read to a length of 250 bp along the sequencing direction using *MEDIPS* v.1.10.0 (*extend=250, uniq=F, window_size=50, BSgenome=‘BSgenome.Hsapiens.UCSC.hg19'*), and the resulting coverage profiles (normalized reads counts, RPKM) were presented. Further, to calculate fold change in H3K27ac enrichment levels between carriers of risk and non-risk allele at genomic regions harbouring the SNPs of interest (shown in Fig. 3c), we used the *MEDIPS* bioinformatics pipeline (as above) to obtain normalized reads counts for a 200 bp region around the SNP site (see Supplementary Data set 4). Genome-wide analysis of this entire data set will be reported in a separate manuscript (under preparation).

### CTCF-ChIP and analysis of allele-specific binding

Purified chromatin from 10 × 10^6^ polarized T_H_1 and T_H_2 cells (31 samples from 11 donors) and 1 × 10^6^ primary CD8^+^ T cells (13 samples from 9 donors) were immunoprecipitated with a polyclonal anti-CTCF antibody (Lot #2142232; 07-729; EMD Millipore), resulting in a total of 44 assays (including 13 technical duplicates). Chromatin was incubated with 10 μl or 5 μl antibody solution (for 10 × 10^6^ cells or 1 × 10^6^ cells ChIP, respectively), pre-coated with protein A-coated magnetic beads (Invitrogen). Immunocomplexes were captured, washed, and eluted and DNA was purified as described above. DNA sequences from control and target sites were quantified by real-time PCR (see Supplementary Table 1) to assess enrichment due to CTCF binding. Data are presented as fold enrichment over control region, which has previously been shown to not bind CTCF^61^. The donors were categorized (into homozygous risk, heterozygous and homozygous non-risk) based on allelic status of SNP overlapping the respective CTCF motif as shown in Fig. 4a and Supplementary Data set 3.

For allele-specific analysis, purified chromatin from input and CTCF-ChIP samples were amplified by PCR using primers that cover the CTCF-binding site overlapping SNPs rs12936231 (G/C; *ZPBP2* site) and rs4065275 (A/G; *ORMDL3* site), and analysed using the Sanger sequencing. Using MacVector, chromatogram traces of donors heterozygous for asthma risk SNPs (as above) were analysed to determine allele-preference for CTCF. The ratio of each allele was calculated using the signal intensity of each base at the position of the respective SNP. Data are shown as percentage of DNA sequences containing the risk and non-risk SNPs at rs12936231 (*ZPBP2* site) or rs4065275 (*ORMDL3* site).

### Analyses of DHS and overlap with 17q21 SNPs

To define the collection of 17q21 asthma-risk SNPs, we first downloaded all asthma-associated SNPs from the databases of GWAS Integrator^62^ and HaploReg v3 (ref. 63) (August 2015), yielding a total of 96 significant lead SNPs (*P*<1.0 × 10^−5^) distributed over 75 loci (with 6 lead SNPs in the 17q21 locus). SNPs in tight genetic linkage (*r*^2^>0.8) were retrieved based on data from the Phase I of the 1,000 genome project using European (EUR) as reference population^64^ (calculations performed using HaploReg v3, Supplementary Data set 1). Among the total of 3,502 asthma-associated SNPs (lead SNPs+linked SNPs), 136 SNPs were identified in the asthma-susceptibility locus on chromosome 17 (17q21).

To determine the number of DHS in the asthma loci, we downloaded genomic annotations of DHS from 64 primary cells types, 21 fetal tissues and 28 cell lines provided by the ENCODE Project Consortium (ENCODE Encyclopedia, version 2; http://www.encodeproject.org)^31^ and the NIH Epigenomics Roadmap Consortium (http://www.roadmapepigenomics.org)^32^, and generated by the labs of Stamatoyannopoulos (University of Washington) and Crawford (Duke University). We utilized processed data provided by the ENOCDE project; here the Stam lab merged all DNase peak data from the Stam and Crawford labs. This merging process formed one combined DNase-Seq data set with non-overlapping DHS. The Stam lab then identified the ‘master' peak in each region, defined as the peak in the region with highest peak height/z-score. Utilizing this data set, loci were defined by the location of the respective SNPs extended for 10 kb at each end. We found a total of 462 unique DHS in the 17q21 asthma-risk locus (chr17: 37,899,254–38,139,253 (hg19); 240 kb).

Next, to determine the average number of DHS in all loci for each cell type, we merged replicates from cell types of similar origin (see details in Supplementary Data set 2a). Of note, merged tracks of mixed or parental populations (for example data sets on ‘CD3 cells' or ‘CD4 primary cells') were excluded when data on subsets (such as ‘CD4 naive cells', ‘T_H_1 cells' and ‘CD8 cells') were available. Our merged data set was used to determine the number of DHS within each cell type or tissue group (shown in Fig. 1d and Supplementary Fig. 1b) and to identify DHS that intersect directly with 17q21 SNPs (shown in Fig. 1e,f). The full list of merged DHS, including the number of single tracks for each cell type or tissue group, and the distribution of DHS in all asthma-associated loci (each defined by the location of the respective SNPs extended for 10 kb at each end) are provided in Supplementary Data set 2.

To predict the functional SNPs in the 17q21 locus, we performed motif scan analysis for the genomics regions highlighted in Fig. 3a using FIMO at the default parameters (*P*<1 × 10^−4^), implementing the motif models from the JASPAR database^65^ (see details Supplementary Data set 5).

### 4C-Seq assay and analysis

The 4C-Seq assay was used to determine regions interacting with the *ORMDL3* promoter region^40^,^41^. Briefly, naive CD4^+^ T cells (or HUT-78 cells) were cross-linked and flash frozen. Cells were lysed, chromatin was digested using the 4-base cutter DpnII and restricted ends re-ligated in situ as described^36^. The chromatin was de-crosslinked and digested using the 4-base cutter MseI (or NlaIII) in order to produce small products^40^. Following a second ligation step in a large reaction volume (to minimize inter-molecular ligations), DNA sequences containing the *ORMDL3* promoter region (anchor point/bait) were amplified by PCR (see Supplementary Table 1) and sequenced to generate 1–2 million 150 bp single-end reads per sample. Conditions were initially optimized in HUT-78 cells using five different baits located in the *ORMDL3* promoter region (baits A-E), and bait D, which showed most interactions, was chosen to perform the 4C-Seq assay in primary CD4^+^ T cells.

Raw fastq files were filtered to remove reads that do not contain the expected bait sequence. From the remaining reads, the bait sequence was removed and reads were mapped against the genomic sequence (hg19) using either Bowtie 1.1.2 (4C-ker^42^) or the custom 4Cseqpipe^40^ mapper (perl 4cseqpipe.pl -map). Number of reads per each restriction enzyme fragment was computed using coverageBed (bedtools) on the fragmented genome (Supplementary Data set 7). Regions with significant interactions were calculated using 4C-ker (near-cis function with *K*=5). Differentially interacting regions at an adjusted *P* value of 0.1 were called using the differential analysis function of the 4C-ker tool (Supplementary Data set 6). All domainograms (for example, Fig. 5c) were generated using the 4Cseqpipe tool (near-cis parameter; trend resolution of 5,000; using mean as the statistics).

### Cell culture and stimulation

For siRNA knockdown studies memory CD4^+^ T cells were cultured in Iscove's Modified Dulbecco's Medium (Invitrogen) supplemented with 5% (vol/vol) heat-inactivated fetal bovine serum and 2% (vol/vol) human AB serum (CellGro) with recombinant human IL-7 (5 ng ml^−1^; Miltenyi Biotec). For *in vitro* differentiation of naive cells into T_H_1 or T_H_2 cells, FACS-sorted naive CD4^+^ T cells were cultured with Human T-Activator CD3/CD28 Dynabeads (Invitrogen) at a bead-to-cell ratio of 1:1 in the presence of recombinant human IL-12 (rhIL-12; 5 ng ml^−1^) and anti-IL-4 antibody (5 μg ml^−1^), or recombinant human IL-4 (rhIL-4; 10 ng ml^−1^) and anti-IFN-γ antibody (10 μg ml^−1^), respectively. Recombinant IL-4, IL-12 and anti-IL-4 antibody were from R&D Systems; the anti-IFN-γ antibody was obtained from BD Pharmingen. After 48 h of culture, the anti-CD3/CD28 Dynabeads were removed and the cells were expanded in culture with recombinant human IL-2 (rhIL-2; 100 IU ml^−1^; National Cancer Institute). After 6 days, cells were washed and analysed for gene expression by real-time PCR. Memory CD4^+^ T cells were intracellularly stained for cytokine detection after stimulation with anti-CD3/CD28 Dynabeads for 6 and 18 h. Brefeldin A (5 μg ml^−1^; Sigma-Aldrich) was added for the final 2 h of culture. The intracellular staining assay was performed as previously described^60^. Data were acquired on a LSR-II (Becton Dickinson) and analysed using FlowJo software (Tree Star).

### siRNA knockdown studies

Memory CD4^+^ T cells were transiently transfected with 0.5 nmol (per 1 × 10^6^ cells) of siRNA pools specific for *ORMDL3*, *GSDMB*, *TBX21*, and *GATA3* or non-targeting siRNA (all ON-TARGETplus SMARTpools from Dharmacon) using the Neon Transfection System (Invitrogen) according to the manufacturer's protocol (settings: 2,200 V, 10 ms, 3 pulses). All siRNA sequences are provided in Supplementary Table 3. Knockdown efficiency was analysed 48 h after transfection by real-time PCR for transcript levels, and by intracellular FACS assay for protein levels when specific and good antibodies were available for ORMDL (ab107639) as previously described^45^, T-bet (4B10) and GATA3 (L50-823) (Fig. 6b and Supplementary Fig. 6a,b); we also verified the effects of *ORMDL3* and *GSDMB* siRNA pools by using the four independent siRNAs in the SMARTpool and the corresponding C911 mismatch control siRNAs^66^, where the nucleotide sequence is altered at bases 9–11, the seed sequence needed for specific targeting (Supplementary Fig. 6e,f and Supplementary Table 3). Viability of cells was analysed 48 h after transfection by using FACS-based Annexin-V and 4′,6-diamidino-2-phenylindole (DAPI) staining. Subsequently, the memory CD4^+^ T cells were stimulated with anti-CD3/CD28 Dynabeads and cytokine production was analysed by real-time PCR and intracellular FACS at various time points. Cytokine secretion after 48 h of stimulation was measured with V-PLEX Validated Assay Kits (Meso Scale Discovery) following the manufacturer's instructions. All data are presented as mean of biological duplicates.

### Data availability

The authors declare that the data supporting the findings of this study are available within the paper (and its supplementary information files).

## Additional information

**How to cite this article**: Schmiedel, B. J. *et al*. 17q21 asthma-risk variants switch CTCF binding and regulate IL-2 production by T cells. *Nat. Commun.* **7**, 13426 doi: 10.1038/ncomms13426 (2016).

**Publisher's note:** Springer Nature remains neutral with regard to jurisdictional claims in published maps and institutional affiliations.

## Supplementary Material

Supplementary Information Supplementary Figures 1-6, Supplementary Tables 1-3 and Supplementary References

Supplementary Data 1 GWAS-derived asthma-associated lead SNPs (highlighted in gray) and SNPs in tight genetic linkage.

Supplementary Data 2 Supplementary Dataset 2A: Analysis of publically available DNase-Seq datasets (ENCODE Project Consortium; see Methods), DNase hypersensitivity sites (DHS) in 17q21 locus in primary cell types (n=212), fetal tissues (n=279) and cell lines (n=107); in alphabetical order. Name of cell type/tissue, sample title (as obtained from ENCODE master file) of unique tracks used for the grouping, total number of DHS genome-wide and in the 17q21 locus of each track. Supplementary Dataset 2B: DNase hypersensitivity sites (DHS) in 17q21 locus in primary cell types (immune cell types used in analysis (n=10) printed in bold), fetal tissues and cell lines; ordered by number of DHS in 17q21 locus. Number of unique tracks used for merging cells and tissue of similar origin, average number of DHS genome-wide and in the 17q21 locus, and number of DHS overlapping with 17q21 SNPs (Fig. 1). Supplementary Dataset 2C: Average number of DNase hypersensitivity sites (DHS) in asthma-associated loci (n=75) in primary cell types (immune cell types used for analysis (n=10) printed in bold), fetal tissues and cell lines (see Methods). Loci are named by a prominent lead SNP and ordered by the calculated ratio of immune versus non-immune cell types; cell types in alphabetical order.

Supplementary Data 3 Age and gender of study subjects, along with their classification based on the genotype for rs7216389. Genotype status of other linked SNPs (rs12936231, rs4065275) that overlap CTCF binding sites is also shown.

Supplementary Data 4 Normalized counts for H3K27ac enrichment (RPKM) in 400 bp window around the 17q21 SNPs of interest (200 bp region on either side; see Methods) for homozygous risk (rs7216389 T/T, n=36) and non-risk (rs7216389 C/C, n=7) samples. The P value was calculated using the Mann-Whitney U test.

Supplementary Data 5 Prediction of TF binding sites affected by 17q21 SNPs. List of 17q21 asthma-risk SNPs overlapping DHS (described in Fig. 3a) that affect transcription factor (TF) binding motifs. The list shows the respective 17q21 SNPs (for asthma-risk and non-risk allele), the name of the TF along with its motif (the nucleotide of the 17q21 SNP is illustrated in bold font), the motif scores, P values from FIMO analysis. Missing entries indicate that the predicted TF binding site was only found on one allele by the FIMO analysis (using default settings).

Supplementary Data 6 Normalized counts and P values from the 4C-Seq analysis of the ORMDL3 promoter region (chr17: 37,849,238 - 38,189,238 (hg19); 340 kb) using the 4C-ker method, ordered by adjusted P values. Significantly differing regions are highlighted in red (up in risk) and blue (up on non-risk).

Supplementary Data 7 Raw read counts from 4C-Seq analysis of the ORMDL3 promoter region (chr17: 37,849,238 - 38,189,238 (hg19); 340 kb), ordered by chromosomal location. Data were filtered to include only regions with a sum of >50 reads across all donors. Regions enriched in at least 3 of 4 donors for risk or non-risk alleles are highlighted (red or blue, respectively).
