# Supplementary material for: 17q21 asthma-risk variants switch CTCF binding and regulate IL-2 production by T cells
Source: Nat Commun. 2016 Nov 16;7:13426. doi: 10.1038/ncomms13426 (PMC5116091; doi:10.1038/ncomms13426)
Supplement: Supplementary Information — Supplementary Figures 1-6, Supplementary Tables 1-3 and Supplementary References [file ncomms13426-s1.pdf]

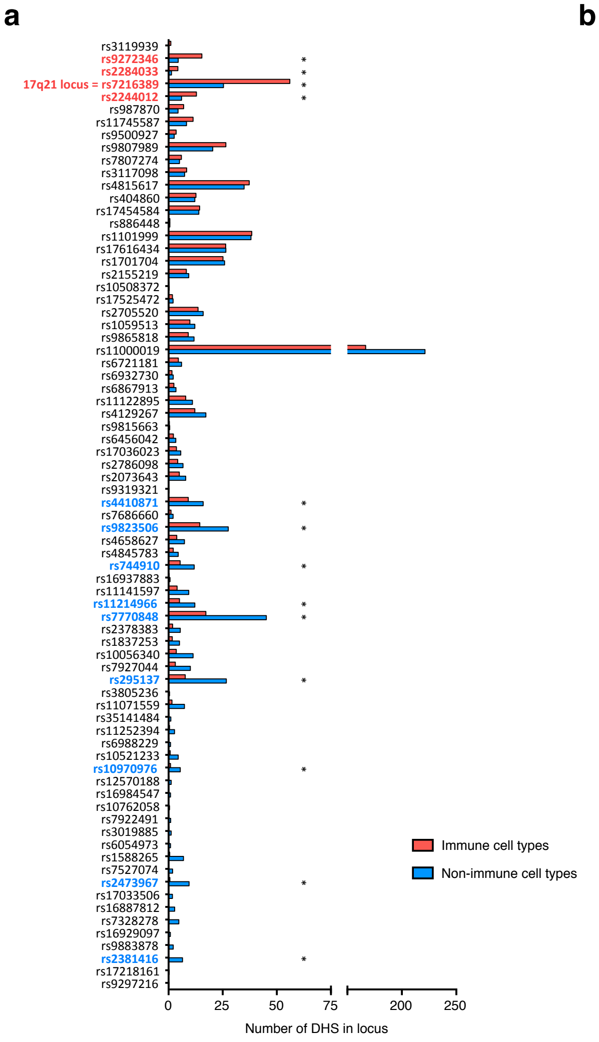

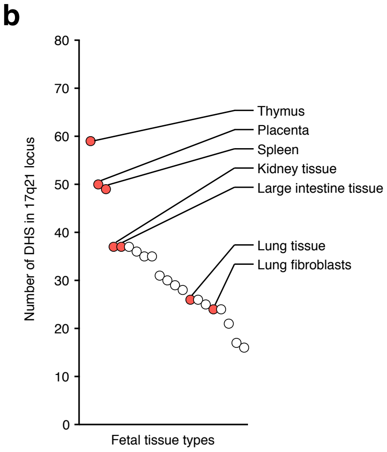

Supplementary Figure 1. DNase hypersensitivity sites (DHS) in fetal cell types. (**a**) Graph shows the average number of DHS in asthma-associated gene loci (n=75) of immune versus non-immune cell types (n=10 and n=52, respectively). The loci are labeled by a prominent lead SNP (full list in Supplementary Dataset 1). Marker SNPs for loci that were enriched for DHS in immune or non-immune cell types are bolded and colored in red or blue, respectively (full list in Supplementary Dataset 2c); *$P < 0.001$ by Student's paired two-tailed t test, and following Bonferroni correction for multiple testing. (**b**) Graph shows the 21 fetal cell types (indicated as dots, profiled by the ENCODE Project Consortium, see Methods) ordered based on the number of DHS in the 17q21 locus. The top hits and discussed cell types are labeled and marked in red (full list in Supplementary Dataset 2b).

a

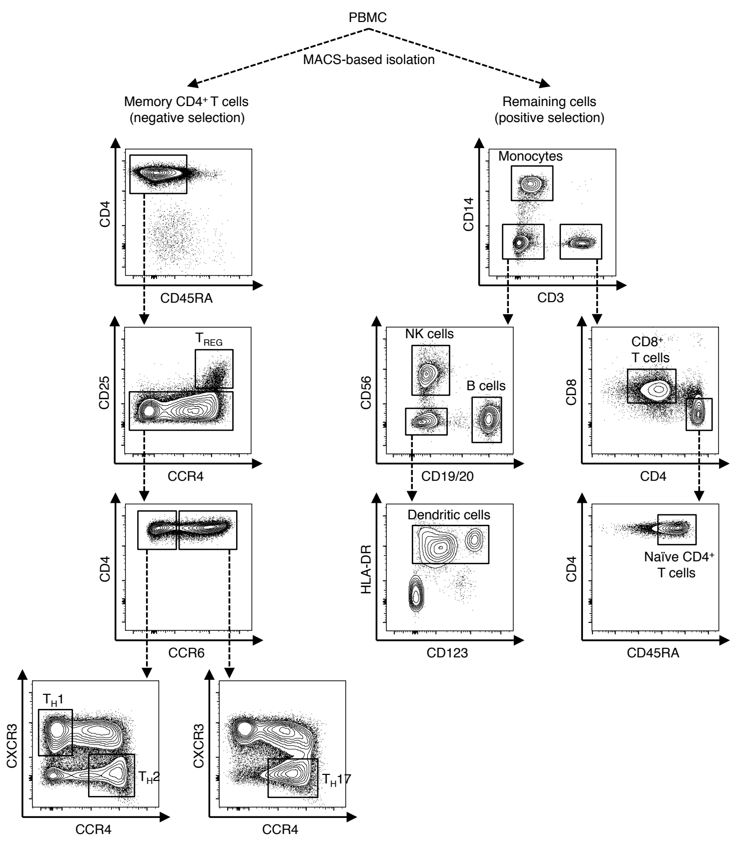

b

Cell type	Surface markers
Naïve $CD4^{+}$ T cells	$CD3^{+}$ $CD4^{+}$ $CD45RA^{+}$
T_H1 cells	$CD4^{+}$ $CD45RA^{-}$ $CCR6^{-}$ $CXCR3^{+}$ $CCR4^{-}$
T_H2 cells	$CD4^{+}$ $CD45RA^{-}$ $CCR6^{-}$ $CXCR3^{-}$ $CCR4^{+}$
T_H17 cells	$CD4^{+}$ $CD45RA^{-}$ $CCR6^{+}$ $CXCR3^{-}$ $CCR4^{+}$
T_{REG} cells	$CD4^{+}$ $CD45RA^{-}$ $CCR4^{+}$ $CD25^{+}$
$CD8^{+}$ T cells	$CD3^{+}$ $CD8^{+}$
B cells	$CD3^{-}$ $CD19^{+}$ $CD20^{+}$
NK cells	$CD3^{-}$ $CD56^{+}$
Monocytes	$CD14^{+}$
Dendritic cells	$CD3^{-}$ $CD14^{-}$ $CD19^{-}$ $CD20^{-}$ $CD56^{-}$ (lin^{-}) $HLA\text{-}DR^{+}$

Supplementary Figure 2. Cell sorting strategy. (**a**) FACS plots describing the sorting strategy used for isolating immune cell types from peripheral blood mononuclear cells (PBMC). (**b**) Surface markers used for sorting the indicated cell types; the strategy for isolating T cell subsets has been described previously[1].

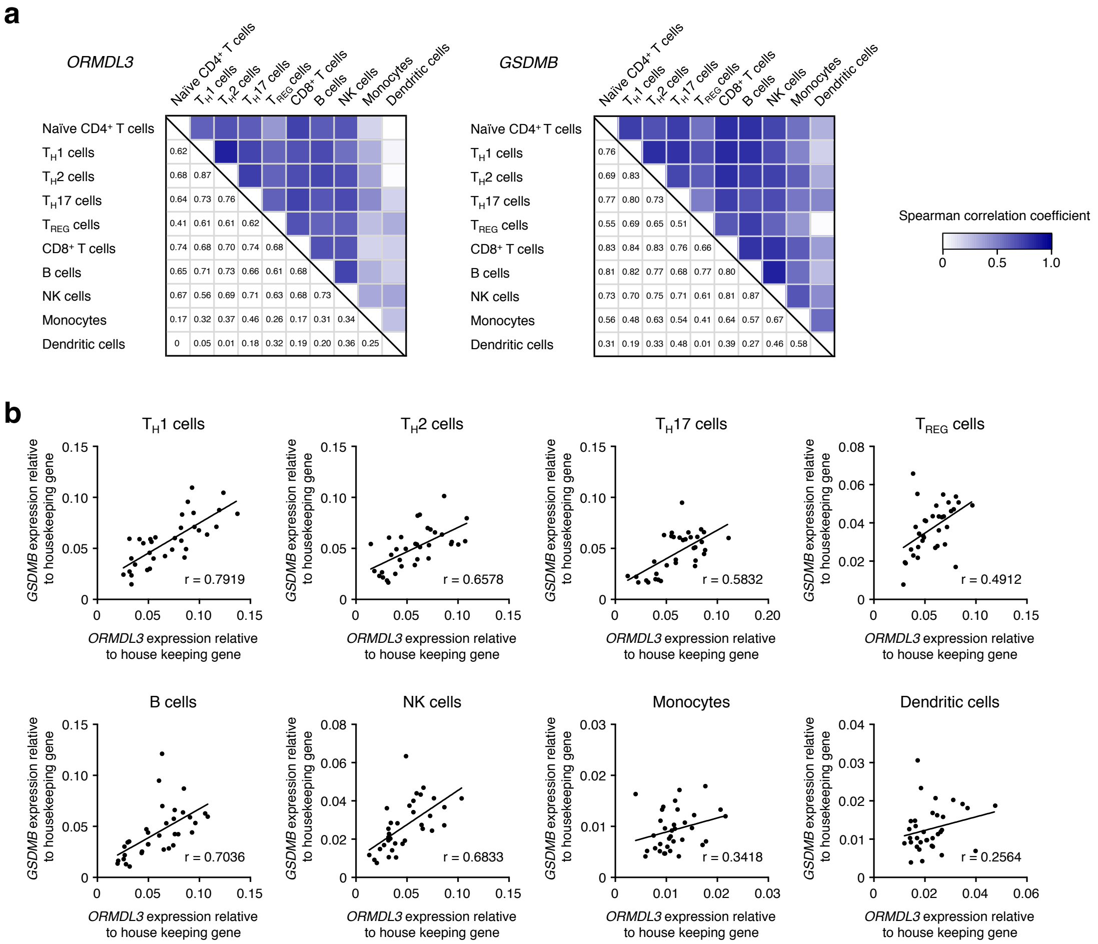

Supplementary Figure 3. Correlation of *ORMDL3* and *GSDMB* transcript levels. (**a**) Heat maps showing the correlation of *ORMDL3* (left panel) and *GSDMB* (right panel) transcript levels among the indicated 10 immune cell types (n=34 donors); numbers show the Spearman correlation coefficient value (r) for each comparison (range: *ORMDL3*, 0.0017 to 0.8738; *GSDMB*: 0.0135 to 0.8717). (**b**) Plots showing correlation between *ORMDL3* and *GSDMB* transcript levels in the indicated cell types (n=34 donors); r value indicates the Spearman correlation coefficient.

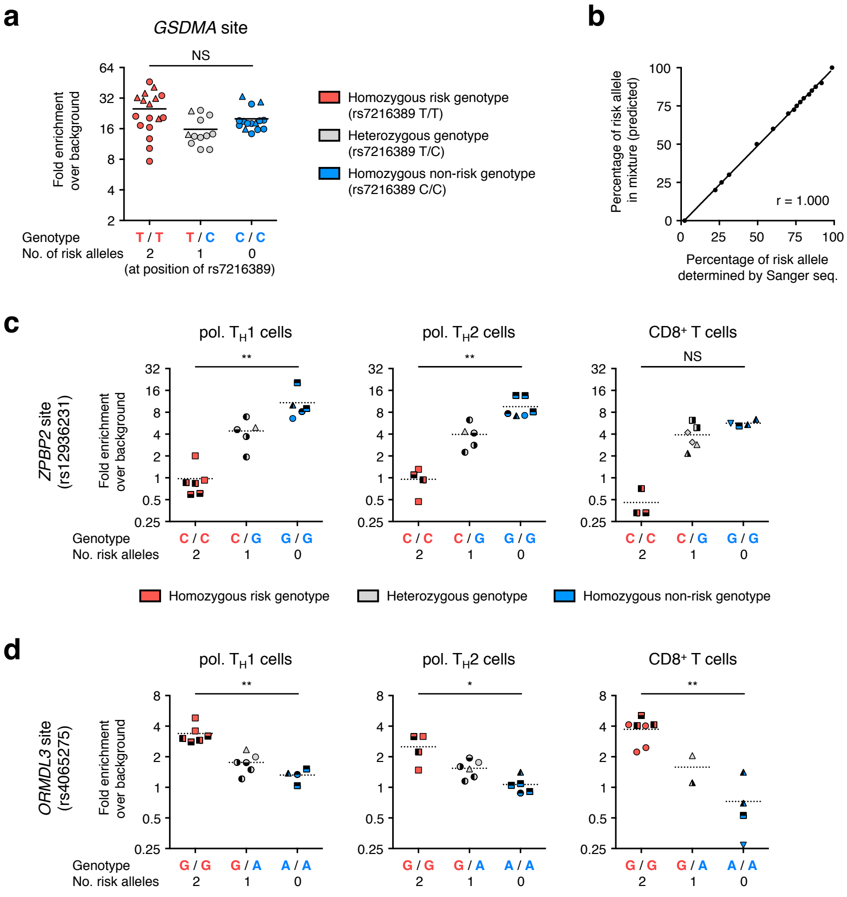

Supplementary Figure 4. 17q21 SNPs modulate binding motifs of CTCF. (**a**) Real-time PCR quantification of an invariant CTCF site 3' of *GSDMA* after anti-CTCF ChIP of chromatin extracts obtained from polarized T_H1, T_H2 and primary CD8$^+$ T cells (see Methods). Due to the lack of a 17q21 SNP in the binding motif, donors were categorized based on allelic status of SNP rs7216389 (~65 kb upstream of the *GSDMA* site). Data are expressed as fold enrichment relative to an irrelevant background control (see Methods). Data were obtained from four independent experiments; NS, not significant by Mann-Whitney U test. (**b**) Correlation between the expected percentage of DNA sequences containing the risk SNP, obtained by pre-mixing of chromatin of risk and non-risk donors in various ratios (y-axis), and its measured values by Sanger sequencing (x-axis, see Methods). (**c,d**) Data sets of CTCF ChIP on *ZPBP2* site (rs12936231) or *ORMDL3* site (rs4065275), as shown in Fig. 4b, re-organized to indicate biological and technical duplicates from polarized T_H1 (left panels), T_H2 cells (middle panels) and primary CD8$^+$ T cells (right panels). Each data set from a single donor is represented by a unique point; n=44 assays from 15 subjects (see Methods).

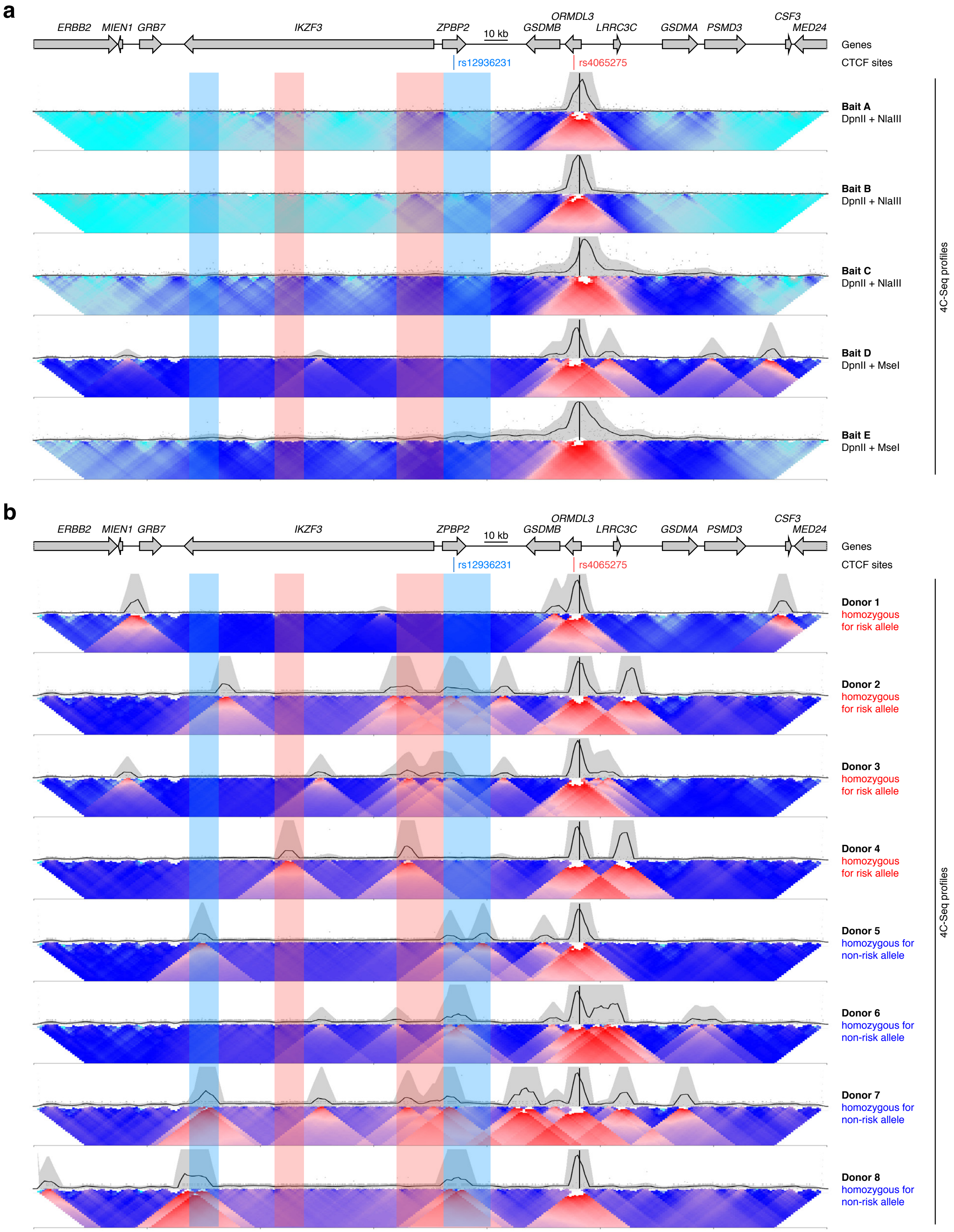

Supplementary Figure 5. 4C-Seq assay on the *ORMDL3* promoter region. UCSC gene tracks of the 17q21 locus (chr17: 37,849,238 - 38,189,238 (hg19); 340 kb) showing CTCF-binding sites that overlap with the linked SNPs rs12936231 (C/G; *ZPBP2* site) and rs4065275 (G/A; *ORMDL3* site). 4C-Seq domainograms generated using 4Cseqpipe[2] (see Methods) are displayed for (**a**) the 5 different baits (A-E) on HUT-78 cells and (**b**) the single donors of CD4+ T cells from subjects homozygous for the risk (donors 1-4) and non-risk alleles (donors 5-8) (bait D), merged data shown in Fig. 5c. The bait region (*ORMDL3* promoter) is marked as a black line. Shaded boxes highlight regions that interact with the bait region at the *ORMDL3* promoter in the risk and non-risk alleles (shown in red and blue color, respectively).

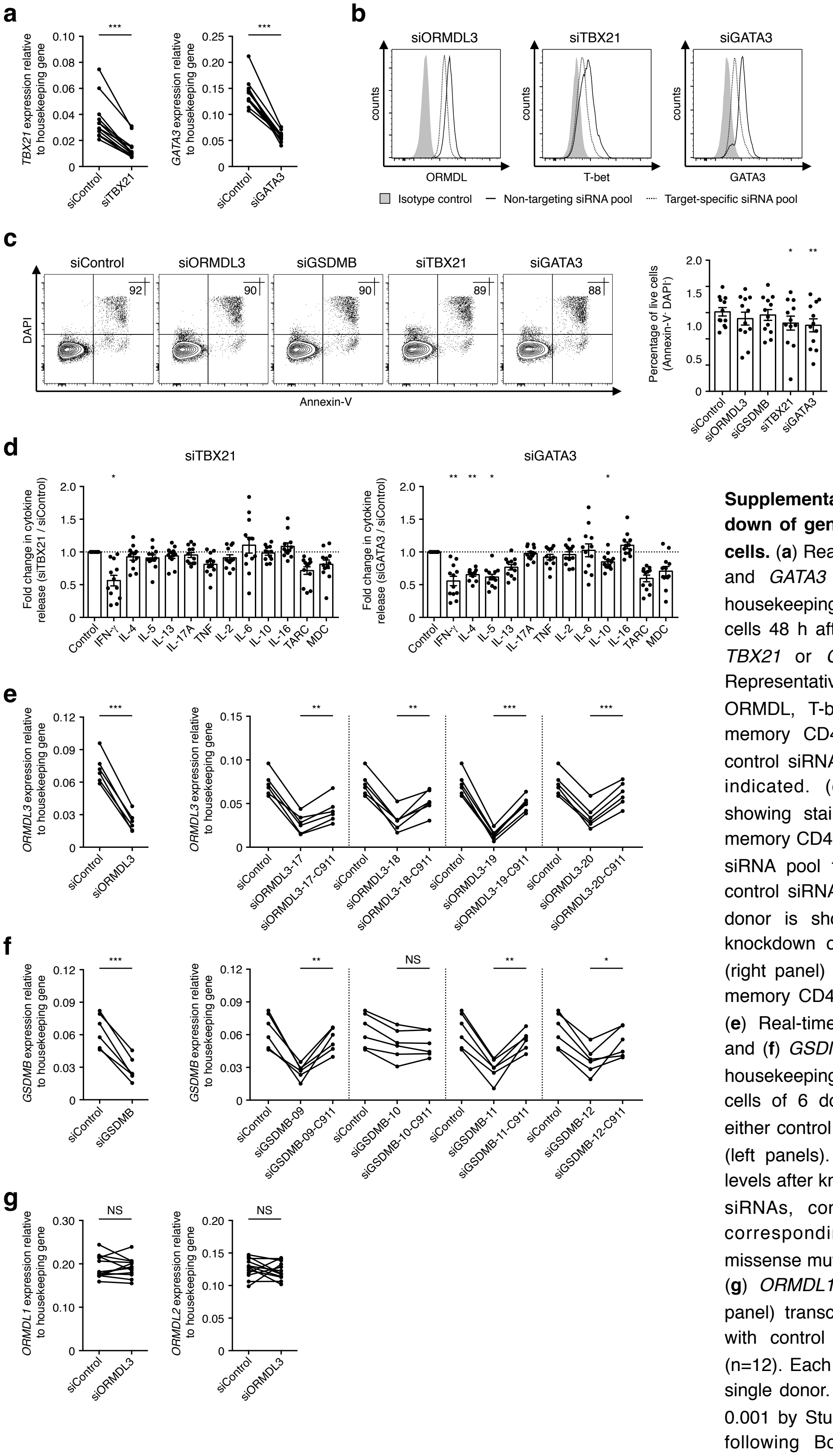

Supplementary Figure 6. Effects of knock-down of genes of interest in primary CD4⁺ T cells. (**a**) Real-time PCR quantification of *TBX21* and *GATA3* transcript levels (relative to the housekeeping gene *YWHAZ*) in memory CD4⁺ T cells 48 h after knockdown with control siRNA, *TBX21* or *GATA3* siRNA pools (n=12). (**b**) Representative histograms of FACS staining for ORMDL, T-bet and GATA3 protein levels in memory CD4⁺ T cells after knockdown with control siRNA or gene-specific siRNA pools as indicated. (**c**) Representative FACS plots showing staining for Annexin-V and DAPI in memory CD4⁺ T cells 48 h after knockdown with siRNA pool for the indicated target genes or control siRNA; percentage of live cells in each donor is shown to the right. (**d**) Effects of knockdown of *TBX21* (left panel) and *GATA3* (right panel) transcripts on cytokine release by memory CD4⁺ T cells, as described in Fig. 6c. (**e**) Real-time PCR quantification of *ORMDL3* and (**f**) *GSDMB* transcript levels (relative to the housekeeping gene *YWHAZ*) in memory CD4⁺ T cells of 6 donors 48 h after knockdown with either control siRNA or target gene siRNA pools (left panels). *ORMDL3* and *GSDMB* transcript levels after knockdown with the respective single siRNAs, contained in the pool, and each corresponding C911 control[3] including a missense mutation, are shown in the right panel. (**g**) *ORMDL1* (right panel) and *ORMDL2* (left panel) transcript levels 48 hr after knockdown with control siRNA or *ORMDL3* siRNA pool (n=12). Each dot or pair represents data from a single donor. **P* < 0.05, ***P* < 0.01, and ****P* < 0.001 by Student's paired two-tailed t test, and following Bonferroni correction for multiple testing in **d** (see Methods).

Supplementary Table 1. Primer sequences for genotyping, analysis of gene expression, analysis of CTCF ChIP, Sanger sequencing assay and 4C-Seq.

Gene / target site	Forward primer (5' - 3')	Reverse primer (5' - 3')	Product size (bp)
rs12936231 *	CTTACATTAGCCCCCAGATG	TGCAGGCACATGTTTAGTCC ***	107
rs4065275	TCTGAAGCATGGAAAGTGGA ***	GGAGTGGGTAGCATCACAAG	641
rs7216389	CAGTTCTGTCGCTGTTGTTTG	CAAGAAGCAAATGGTCCCTAA ***	377
YWHAZ	CCAATGCTTCACAAGCAGAGAGCA	ATCCCTTTCTTGTCATCACCAGCG	103
ORMDL1	CTGACCAGGGTAAAGCAAGG	TGTGGCATTTTGGGAATTAG	199
ORMDL2	ATGGACTATGGGCTCCAGTTT	AATGAGGCTGTGTTGATGAGG	128
ORMDL3	CCAACCTCATTCACAACATGGGCA	GGTGTGATGGTCAAGAACTTCCGA	163
GSDMB	GATGCCCTGCTAGAGCTGTC	CACAGAGAATTCGTGCCTCA	172
TBX21	GACTCCCCCAACACAGGAG	GGAGGGACTGGAGCACAAT	121
GATA3	GAACTGTCAGACCACCACAA	GCCTTCCTTCTTCATAGTCAGG	127
IL2	CTCACCAGGATGCTCACATTTA	CCTCCAGAGGTTTGAGTTCTTC	97
chr11:1,983,833-1,983,994 ** (negative control)	GAGCTCTAAGGGAGGCTCC	CATCATGGTGTCCTCACAGG	162
ZPBP2 site (at rs12936231) *	CTTACATTAGCCCCCAGATG	TGCAGGCACATGTTTAGTCC	107
ORMDL3 site (at rs4065275)	ATGAGGACCCTCTGCCGACAA	CCACATTAGGCCTTCAGTGAGACA	91
GSDMA site	ACCTTAATTGACCACCAGAGGGAG	CCTTGTAAGGACGCTTCAGGTCAA	126
ZPBP2 site (at rs12936231) *	CTTACATTAGCCCCCAGATG	TGCAGGCACATGTTTAGTCC ***	107
ORMDL3 site (at rs4065275)	ATGAGGACCCTCTGCCGACAA	GTGAGTGGATGCAGTGACCTCAAA ***	194
4C *ORMDL3* promoter (bait A)	AAGAGGAGCACAAAACATCCA	TGTTTCCTCATTGCCAGTCTT	-
4C *ORMDL3* promoter (bait B)	GGCCTCTTGGTTCCTTCC	GGGCCAAAGTTGAGAGGTTAT	-
4C *ORMDL3* promoter (bait C)	CAAGGGCACAGACTTTACTGC	GGTGCAGTGGAAAAATCCTG	-
4C *ORMDL3* promoter (bait D)	TCATAACCTGCCTCAAACCA	TGTTTCCTCATTGCCAGTCTT	-
4C *ORMDL3* promoter (bait E)	GGCCTCTTGGTTCCTTCC	GGCCCGGATATTTGTATGTG	-

* Primer sequences were obtained from Verlaan et al. Am J Hum Genet. 2009.

** Primer sequences were obtained from Wendt et al. Nature. 2008 (non-CTCF binding site in ICR of H19/IGF2 locus on chr.11).

*** Specific primer used in Sanger sequencing reaction (*e.g.* for genotyping).

Supplementary Table 2. Antibodies and reagents employed in FACS analysis.

Antigen / Dye	Conjugate	Clone	Supplier
CD3	PE-Cy7	UCHT1	Biolegend
CD4	APC-Cy7	RPA-T4	Biolegend
CD8	FITC	RPA-T8	Biolegend
CD14	BV 421	M5E2	Biolegend
CD19	FITC	HIB19	Biolegend
CD20	FITC	2H7	Biolegend
CD25	FITC	M-A251	BD Biosciences
CD45RA	Alexa Fluor 700	HI100	Biolegend
CD56	APC	HCD56	Biolegend
CD123	PE	6H6	Biolegend
CXCR3 (CD183)	PerCP-Cy5.5	1C6/CXCR3	BD Biosciences
CCR4 (CD194)	PE	205410	R&D Systems
CCR6 (CD196)	Biotin	11A9	BD Biosciences
HLA-DR	PerCP-Cy5.5	G46-6	BD Bioscienes
Streptavin	BV 605	-	Biolegend
Annexin-V	APC	-	BD Biosciences
DAPI	-	-	Life Technologies
Fixable Viability Dye	eFluor506	-	eBioscience
IL-2	PE-Cy7	MQ1-17H12	Biolegend

Supplementary Table 3. Sequences of siRNAs to knockdown expression of *ORMDL3, GSDMB, TBX21, GATA3* and the corresponding C911 controls (the missense mutation is printed in bold).

siRNA	Target sequence (sense; 5' - 3')	Dharmacon SMARTpool ID
siORMDL3-17	GAACAUGGACCACGCAGUU	J-017002-17
siORMDL3-18	ACACUAAGUACGACCAGAU	J-017002-18
siORMDL3-19	CGGUACGGCUUCUGGAUUG	J-017002-19
siORMDL3-20	UGGGUAGGGAGCUGUCUAA	J-017002-20
siORMDL3-17-C911	GAACAUGG**UGG**ACGCAGUU	-
siORMDL3-18-C911	ACACUAAG**AUG**GACCAGAU	-
siORMDL3-19-C911	CGGUACGG**GAA**CUGGAUUG	-
siORMDL3-20-C911	UGGGUAGG**CUC**CUGUCUAA	-
siGSDMB-09	GGUCCUGAGCUAUCGAGUA	J-020262-09
siGSDMB-10	CCGAUCAAUUAAUACGAGA	J-020262-10
siGSDMB-11	GUGCUAAACUCCCUCGCUA	J-020262-11
siGSDMB-12	GAAACUCUGGAGACGGUAA	J-020262-12
siGSDMB-09-C911	GGUCCUGA**CGA**AUCGAGUA	-
siGSDMB-10-C911	CCGAUCAA**AAU**AUACGAGA	-
siGSDMB-11-C911	GUGCUAAA**GAG**CCUCGCUA	-
siGSDMB-12-C911	GAAACUCU**CCU**GACGGUAA	-
siTBX21-05	UCCAGUCCCUCCAUAAGUA	J-005217-05
siTBX21-06	GAACUUUGAGUCCAUGUAC	J-005217-06
siTBX21-07	GAGCAGAUGACAUGAUGAA	J-005217-07
siTBX21-08	ACACGCAUAUCUUUACUUU	J-005217-08
siGATA3-06	GUACAGCUCCGGACUCUUC	J-003781-06
siGATA3-07	CCCAAGAACAGCUCGUUUA	J-003781-07
siGATA3-08	GAAGGCAUCCAGACCAGAA	J-003781-08
siGATA3-09	CAUCGACGGUCAAGGCAAC	J-003781-09

Supplementary References

1. Zielinski, C.E., et al. Pathogen-induced human TH17 cells produce IFN-gamma or IL-10 and are regulated by IL-1beta. *Nature* **484**, 514-518 (2012).
2. van de Werken, H.J., et al. Robust 4C-seq data analysis to screen for regulatory DNA interactions. *Nature methods* **9**, 969-972 (2012).
3. Buehler, E., Chen, Y.C. & Martin, S. C911: A bench-level control for sequence specific siRNA off-target effects. *Plos One* **7**, e51942 (2012).
